# Transcriptomics predicts compound synergy in drug and natural product treated glioblastoma cells

**DOI:** 10.1371/journal.pone.0239551

**Published:** 2020-09-18

**Authors:** Lavinia-Lorena Pruteanu, Liliya Kopanitsa, Dezső Módos, Edgars Kletnieks, Elena Samarova, Andreas Bender, Leonardo Dario Gomez, David Stanley Bailey

**Affiliations:** 1 IOTA Pharmaceuticals Ltd, St Johns Innovation Centre, Cambridge, United Kingdom; 2 Department of Chemistry, Centre for Molecular Informatics, University of Cambridge, Cambridge, United Kingdom; 3 Department of Biology, Centre for Novel Agricultural Products, University of York, York, United Kingdom; Columbia University, UNITED STATES

## Abstract

Pathway analysis is an informative method for comparing and contrasting drug-induced gene expression in cellular systems. Here, we define the effects of the marine natural product fucoxanthin, separately and in combination with the prototypic phosphatidylinositol 3-kinase (PI3K) inhibitor LY-294002, on gene expression in a well-established human glioblastoma cell system, U87MG. Under conditions which inhibit cell proliferation, LY-294002 and fucoxanthin modulate many pathways in common, including the retinoblastoma, DNA damage, DNA replication and cell cycle pathways. In sharp contrast, we see profound differences in the expression of genes characteristic of pathways such as apoptosis and lipid metabolism, contributing to the development of a differentiated and distinctive drug-induced gene expression signature for each compound. Furthermore, in combination, fucoxanthin synergizes with LY-294002 in inhibiting the growth of U87MG cells, suggesting complementarity in their molecular modes of action and pointing to further treatment combinations. The synergy we observe between the dietary nutraceutical fucoxanthin and the synthetic chemical LY-294002 in producing growth arrest in glioblastoma, illustrates the potential of nutri-pharmaceutical combinations in targeting this challenging disease.

## Introduction

Glioblastoma multiforme (GBM) is the most common primary and aggressive malignant brain tumor in adults [1], with patients having a median survival after diagnosis of only 12–15 months [1–4]. Current chemotherapy, together with surgery and radiotherapy, provide only minor patient benefit, and there is a considerable need for development of effective new therapies.

One way to achieve efficacious treatment, in particular in areas such as cancer (where multiple disease drivers may exist) and infectious diseases (where mutations are common during treatment, which of course also exists in the case of cancer) is the use of compound combinations [5]. Here in some cases synergy of active ingredients is desired [5], in order to achieve the desired effect at lower dose, which means that the effect of the compound combination is higher than that expected in case of the null hypothesis of additivity. Multiple models exist for this purpose, such as the Loewe model (which assumes additivity of effects), the Bliss model (which assumes independence) and the Highest Single Agent (HSA) model, which only takes the most significant effect into account (as well as other more recent models [6, 7] which in some cases show more intuitive behavior in practice). However, given that the mechanistic reason for synergy is in most cases poorly understood, the model choice is often empirical, which can lead to conclusions that a compound combination is synergistic due to the model chosen, which wouldn’t have been the case with another model (see [7] for a more detailed discussion of this subject). In this work, we employed the Loewe synergy model; however we also applied other models to verify results, and they were found to be in agreement with each other in this case (see results section). Still, generally speaking the identification of synergy of a compound combination depends on the particular definition one chooses for this purpose and this choice is hence of fundamental nature for the analysis of combination screening data.

Our work has characterized the genes and pathways deployed by glioblastoma cells in their response to drugs and natural products. An initial focus on the pro-proliferative phosphatidylinositol 3-kinase/protein kinase B/mechanistic target of rapamycin (PI3K/Akt/mTOR) pathway, has highlighted the key gene expression responses to the well-characterized PI3K inhibitor LY-294002, subsequently uncovering a synergy in growth inhibition between fucoxanthin and LY-294002.

The fact that drug therapy for GBM has been unsuccessful probably reflects our lack of detailed knowledge of the signaling pathways that control GBM growth and differentiation. We know that many key cellular signaling pathways are dysregulated in GBM including proliferation, angiogenesis, components of the mTOR complexes (mTORC) signaling and Nuclear Factor kappa-light-chain-enhancer of activated B cells (NF-kB) signaling [8, 9]. Taken together, these signaling pathway changes constitute a phenotype that may promote tumor survival and thereby contribute to the extreme resistance that this tumor displays towards conventional therapies [10].

LY-294002 targets an important pathway often mutated in GBM, the PI3K/Akt pathway [11], which appears particularly important in glioblastoma proliferation but also plays a central role in the regulation of tumor cell survival, motility, angiogenesis and metabolism [12]. This has led to many attempts to target the PI3K/Akt pathway as a potential treatment option for glioblastoma [13, 14].

PI3K has four catalytic isoforms within class I: phosphatidylinositol 4,5-bisphosphate 3-kinase catalytic subunit alpha isoform (PIK3CA/p110α), phosphatidylinositol-4,5-bisphosphate 3-kinase catalytic subunit beta (PIK3CB/p110β), phosphatidylinositol-4,5-bisphosphate 3-kinase catalytic subunit delta (PIK3CD/p110δ), and phosphatidylinositol-4,5-bisphosphate 3-kinase catalytic subunit gamma (PIK3CG/p110γ). Recent studies implicate overexpression of the PIK3CB/p110β isoform in glioblastoma, where it acts as a selective tumor survival factor. Inhibition of PIK3CB/p110β suppresses viability and growth of GBM cells and xenograft tumors in mice, with minimal cytotoxic effects on normal astrocytes [15].

**Fig 1:**
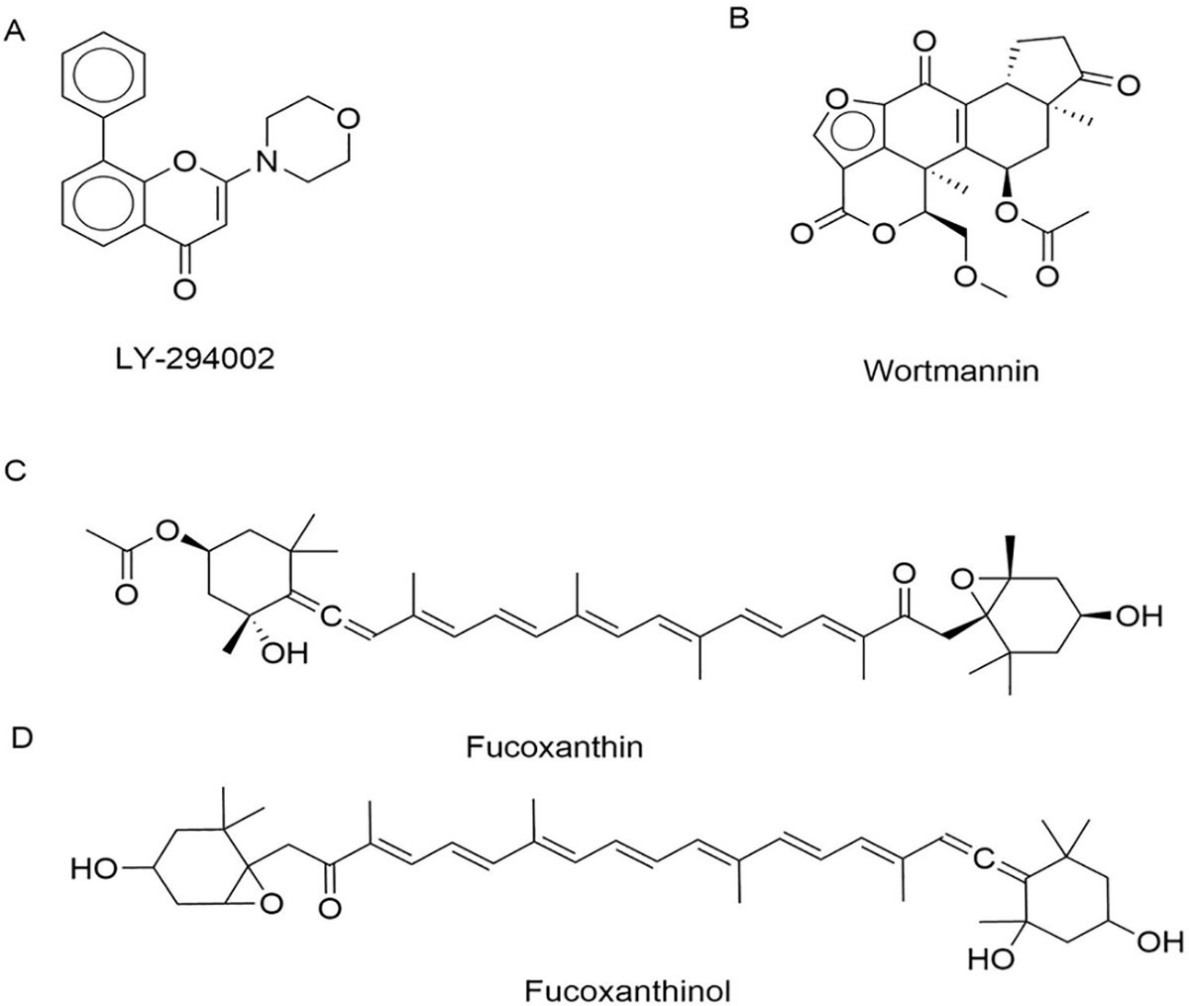
Chemical structures of the PI3K inhibitors discussed in this study. A, LY-294002; B, Wortmannin; C, the natural product fucoxanthin; D, Fucoxanthinol, the deacetylated metabolite of fucoxanthin.

LY-294002 (Fig 1A) was an early PI3K inhibitor to be discovered. LY-294002 is a prototypic, non-selective PI3K inhibitor, which inhibits the ɑ, β and δ isoforms of PI3K, and also has off-target effects on casein kinase 2 (CK2), glycogen synthase kinase 3 beta (GSK3B), mTOR [16, 17]. LY-294002 has previously been shown to enhance the cytotoxicity of temozolomide in U87MG glioma cells by down-regulating genes involved in the PI3K/Akt pathway [18]. For a recent general review of PI3K inhibitors, see [19].

As an earlier alternative to synthetic drugs, natural products targeting PI3K emerged as potential cancer therapeutics: wortmannin (Fig 1B) was isolated as a fungal metabolite, while LY-294002, the first synthetic PI3K inhibitor, was derived from the natural product quercetin [20].

Another natural product with putative PI3K inhibitor activity and therapeutic potential in glioblastoma is fucoxanthin [21–23]. Fucoxanthin is a member of the xanthophyll class of carotenoids, and is present at high concentrations in the brown alga *Saccharina latissimi* [24] where it plays an accessory role in light harvesting and radiation protection.

Fucoxanthin has a particularly interesting and unique molecular structure (Fig 1C), exhibiting antioxidant properties due to a long conjugated backbone characteristic of all carotenoids [25], but possessing an unusual terminal allenic bond and conjugated carbonyl groups [26]. Fucoxanthin has been suggested to act in glioblastoma by suppressing invasion and inducing apoptosis through PI3K/Akt pathway inhibition [17, 27] and Janus Kinase/Signal Transducer and Activator of Transcription (JAK/STAT) pathway inhibition [28, 29].

Previous studies of the activity of fucoxanthin in U87MG glioblastoma cells have shown the modulation of several individual proteins involved in apoptosis, including B-cell lymphoma 2 (Bcl-2), Bcl-2-associated X protein (Bax), caspase-3, and caspase-9, all of which are involved in the PI3K/Akt pathway [30]. In addition, both in hepatocytes and in immortal human cell line (HeLa) cells, fucoxanthin has been shown to stimulate the adenosine monophosphate -activated protein kinase (AMPK) pathway to induce autophagy and cytoprotection [31]. In HeLa cells, fucoxanthin also inhibits Bcl-2, inducing Bax production and caspase-3 cleavage [32], while the deacetylated human metabolite of dietary fucoxanthin, fucoxanthinol (Fig 1D), modulates the NF-κB pathway, caspase activity, Bcl-2 proteins, MAPK, PI3K/Akt, JAK/STAT, activator protein 1 (AP-1), and growth arrest and DNA damage-inducible 45 (GADD45) [33].

These previous studies suggest that fucoxanthin changes the transcriptomic phenotype of cancer cells and warrant detailed analysis of its effects at the molecular and cellular pathway levels. To achieve this effectively and comprehensively, genome-wide approaches are necessary, amongst which gene expression microarray techniques are some of the most robust and powerful [19, 34, 35].

The juxtaposition of phenotypic and target-based drug discovery is yielding a new echelon of pathway-active drugs [36, 37]. Genome-wide gene expression analysis lies at the heart of phenotypic drug discovery, enabling simultaneous high-content pathway exploration and drug response characterization [38, 39]. In cancer, tumor cells seem addicted to certain signaling pathways such as the PI3K/AKT pathway. Not only can the members of signaling pathways be used as potential targets in drug discovery [40], but changes within the pathways themselves can also reveal how cancer cells escape drug treatment. Targeting these escape mechanisms can lead to synergistic therapeutic effects [41, 42].

In the current study, we compare and contrast the effects of fucoxanthin, whose molecular mode of action is incompletely understood, to that of LY-294002, whose activities are comparatively well known [18]. For these studies we have used gene expression analysis in the standard glioblastoma cell line U87MG. We identify effects on the PI3K/Akt/mTOR and other signaling pathways in this glioblastoma model cell system and use the observed gene expression responses of the two compounds to define their potential synergies.

## Materials and methods

### Cell culture and reagents

Human glioblastoma U87MG cells expressing the wild-type *TP53* gene were obtained from the European Collection of Authenticated Cell Cultures (ECACC 89081402) and maintained in DMEM/F12 medium (Gibco, ThermoFisher, UK) supplemented with 10% fetal bovine serum (FBS) (Sigma, UK) and 5% antibiotic/antimycotic solution (Sigma, UK). We have checked U87MG cell line using the ICLAC database (https://iclac.org/databases/cross-contaminations/) and can confirm that this cell line is not in the list of misidentified or contaminated cell lines.

Cells were maintained at 37°C in a humidified atmosphere of 95% air and 5% CO_2_. LY-294002 (L9908, >98% purity) and fucoxanthin (F6932, >95% purity) were purchased from Sigma UK. LY-294002 and fucoxanthin were dissolved in dimethyl sulfoxide (DMSO) before testing.

### Proliferation assay

Cell survival was determined using the Cell Counting Kit-8 (CCK-8) assay (Sigma, UK). U87MG cells were seeded at a density of 8,000 cells/well in 96-well plates and allowed to adhere overnight at 37°C in a humidified atmosphere of 95% air and 5% CO_2_. Fucoxanthin and LY-294002 were tested in triplicate in at least two separate experiments. Dilutions of fucoxanthin and LY-294002 were made first at different concentrations in DMSO and then added to cell medium at a 1:100 ratio. Then, the culture medium was removed from the plates, and fresh medium containing compound was added. Control cells were treated with vehicle solution containing 1% DMSO. Blank controls without cells were also prepared. After 72h treatment, 5 μL of CCK-8 was added to every well containing 100 μL of tested compounds, controls or blank. After 3 h of incubation at 37°C in the dark, the plates were read using a Mithras LB940 multimode microplate reader (Berthold Technologies), and the absorbance values were determined at 490 nm, according to the manufacturer`s instructions. The percentage of surviving cells was calculated for each well using the formula:

$$\%\mathrm{cell}\;\mathrm{survival}=\frac{A_{t}-A_{b}}{A_{c}-A_{b}}\times 100,$$

where A_t_ is absorbance of the medium with tested compound, A_c_ is absorbance of control medium, and A_b_ is absorbance of blank medium.

Concentration-effect relationships for both compounds were analyzed using Prism software version 8.0.1 (GraphPad, Inc., San Diego, CA). Data were fitted using a four-parameter logistic equation.

### Apoptosis assay

The extent of cell apoptosis was measured using the Annexin V-FITC apoptosis detection kit (Abcam, Cambridge, UK). U87MG cells were seeded at a density of 80,000 cells/well in 6-well plates and allowed to adhere overnight at 37°C in a humidified atmosphere of 95% air and 5% CO_2_. Fucoxanthin and LY-294002 were tested at concentrations of 20 μM and 200 μM, their respective IC_50_ values determined in the proliferation assay. Control cells were treated with 1% DMSO (vehicle). After 24h and 48h of treatment, the cells were trypsinized, centrifuged, resuspended in 500 μL of binding buffer followed by the addition of 5 μL Annexin V-FITC and 5 μL propidium iodide (PI) according to the manufacturer's instructions. The samples were incubated at room temperature for 5 min in the dark and then analyzed on a BD Accuri™ C6 Flow Cytometer (BD Biosciences). A total of 10,000 events were counted for each sample. Fluorescence was measured at an excitation wavelength of 480 nm with detection for PI at 530 nm and Annexin V at 585 nm.

### Synergy assay

To study the effects of combined treatments with LY-294002 or fucoxanthin the proliferation assay was performed as described above. Concentration-response relationships for 0.5–500 μM LY-294002 were determined in the presence of 10, 16 or 25 μM fucoxanthin. The experiment was repeated 9 times to ensure statistical significance.

The analysis of combination effects was performed using Combenefit software (version 2.021) [43], primarily with the additive Loewe synergy effect as a baseline model [44], which assumes independent compound mode of actions [7]. However, as described in the introduction, the annotation of synergy of compound combinations is heavily dependent on the definition one uses, and hence also the Bliss and Highest Single Agent (HSA) model were used for comparison (where the main conclusion agreed between methods, see results section for details).

### Microarrays

**Fig 2:**
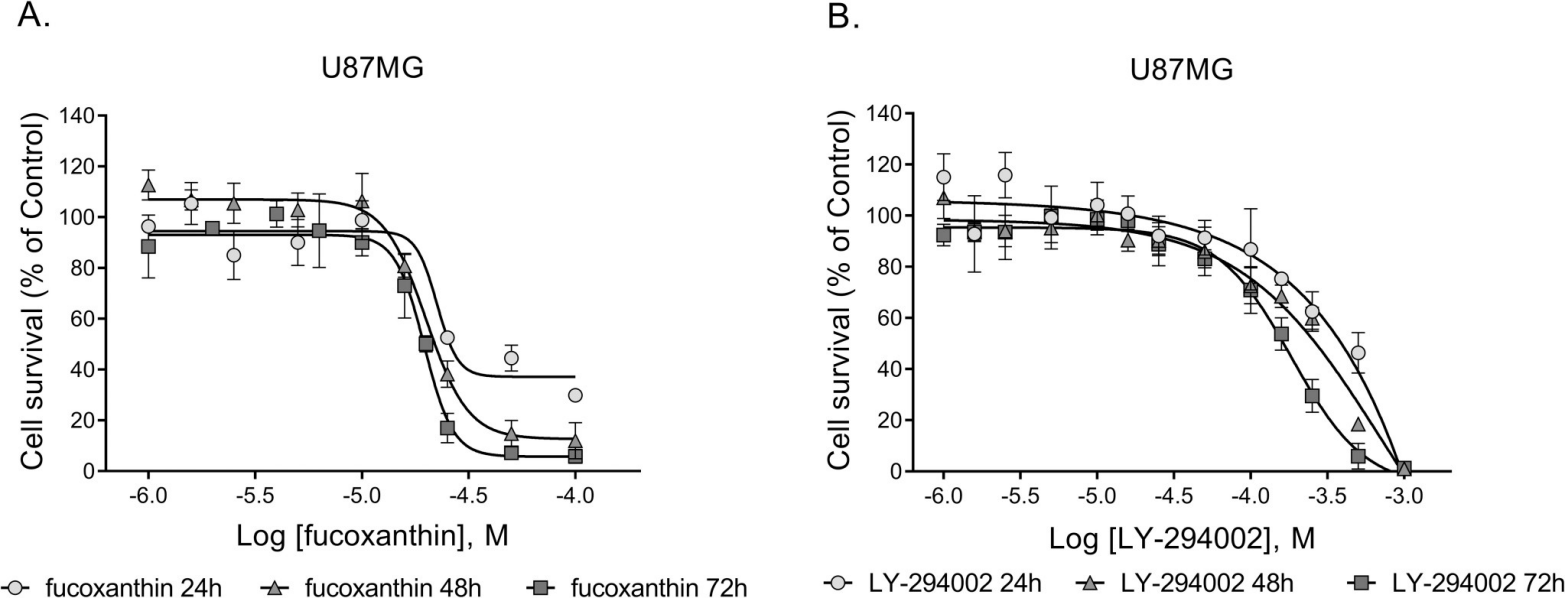
Dose-response relationships of the inhibitory action of fucoxanthin (A) and LY-294002 (B) on U87MG glioblastoma cell survival over 24, 48, or 72h of treatment. Data were normalized relative to the viability of cells treated with vehicle.

Cells were seeded into T25 flasks at a density of 500,000 cells/flask and allowed to adhere and grow for 24h. The culture medium was removed, and fresh medium containing compound for test was added to each flask at the IC_50_ concentration for inhibiting proliferation at 72h, previously determined as 200 μM for LY-294002 and 20 μM for fucoxanthin (Fig 2). Control cells were treated with medium containing 1% DMSO alone. All experiments were performed in triplicate. Cells were visualised during culture using the EVOS Cell Imaging System (Thermo Fisher Scientific, UK).

After 24h and 48h of treatment, the cells were trypsinized, and total RNA was isolated using the RNeasy Mini kit (Qiagen, UK) according to the manufacturer's recommendations. A Thermo Scientific™ NanoDrop instrument was used to quantify the RNA and test the purity of each sample. Aliquots of RNA were frozen at -80°C prior to analysis at ATLAS Biolabs (Berlin, Germany) for microarray experiments.

Total RNA was analyzed by ATLAS Biolabs (Berlin, Germany) using their Affymetrix WT Expression Profiling Standard Service. The quality and quantity of each RNA sample was determined using the Agilent 2100 Bioanalyzer and Nanodrop. For the library preparation (cDNA synthesis, amplification and labeling) GeneChip^®^ WT PLUS reagents were used. 96-array plates were processed on an Applied Biosystems GeneChip 3000 instrument system followed by hybridization, dyeing, washing, and scanning using the GeneChip™ Hybridization, Wash, and Stain Kit from Affymetrix (catalog number: 900720). Hybridization controls, quality control parameters and primary data analysis was performed using Expression Console v1.4 (also from Affymetrix). The analysis was conducted on a Clariom S Human array (Thermo Fisher Scientific, Catalog Number: 902926) with a fixed number of probes per transcript as probe sets consisting of a subset of 10 probes per gene (yielding >20,000 annotated genes, as documented by Affymetrix (www.affymetrix.com/analysis/netaffx/)).

### Microarray data analysis

An expression matrix was created from the average signal intensities by applying the RMA (Robust Multi-array Average) algorithm as a multi-chip model [45]. A control housekeeping gene intron/exon separation area under receiving operating curve value threshold of 0.8 was selected to ensure good sample quality.

Microarray gene expression profiling and visualization were performed using Affymetrix Transcriptome Analysis Console (TAC) software (ThermoFisher) which uses the limma package [46]. In defining differentially expressed genes (DEGs), two criteria were used. Firstly, the probeset absolute log 2 Fold Change (FC) was higher than 1 based on Tukey's bi-weight average between treatment and time matching controls [47], which renders the average value less sensitive to outliers [48]. Secondly, the Benjamini-Hochberg corrected p-value of the ANOVA (Analysis of Variance) test was applied to compare conditions (either time or treatment). We considered a gene to be differentially expressed if it had a corrected p-value lower than 0.05 [49]. The ANOVA tests were calculated with an eBayes (Empirical Bayes Statistics for Differential Expression) variance calculation [50]. Finally, the Clariom S chip’s probe sets were mapped to UniProt SwissProt identifiers [51], keeping only the protein coding genes. If a gene had more than one probe set, the most differentially expressed probe sets were used in all cases.

### Gene pathway analysis

After defining the differentially expressed genes (DEGs), pathway analysis was performed. For each treatment, untreated U87MG cells were used as controls at each corresponding time point. To show the most significantly modulated pathways after compound treatment, WikiPathways map representations [52] were used and the signal intensities of DEGs involved in particular enriched pathways shown as down-regulated or up-regulated in response to specific treatments [53]. Fisher’s Exact Tests were used for the statistical analysis of each component within the pathway.

Gene Set Enrichment Analysis (GSEA) used the Broad Institute GSEA tool [54] version number 3.0, using the Hallmark [55] and the Gene Ontology [56] gene sets from the Broad Institute website at 7th of June 2019. The genes were translated through UniProt to ENTREZ IDs and the genes were ranked based on their fold change.

## Results

### Fucoxanthin and LY-294002 reduce proliferation of U87MG cells

As a first step, we studied the effects of fucoxanthin and LY-294002 on the cellular proliferation of U87MG cells. The compounds were tested at concentrations of 1 to 1000 μM (Fig 2). For both compounds, little effect on cell survival was observed at concentrations of less than 10 μM.

For *in vitro* experiments, we used a 24h cell exposure regime, carried out at drug concentrations equal to the 72h IC_50_ for cell proliferation, determined in earlier dose-response studies with treated and untreated cells. Cell survival decreased at concentrations higher than 10 μM resulting in IC_50_s of 20 μM for fucoxanthin (Fig 2A) and 200 μM for LY-294002 (Fig 2B) after 72h of treatment.

Cellular morphology of U87MG glioblastoma cells after 24 and 48h of treatment with fucoxanthin or LY-294002 clearly differed from that of DMSO-treated control cells (see S1A–S1F Fig). The cells grew as monolayers in the cell culture flasks before treatment, forming some spheres after 24h and 48h. After treatment with fucoxanthin for 24h, most adherent cells were round, with some showing signs of nuclear fragmentation, although cells with pseudopod-like protrusions were also observed. After treatment for 48h, some cells remained adherent, while others floated off their plastic substrates, often as aggregates. The morphology of cells treated with LY-294002 differed from those treated with fucoxanthin, with some cell membrane blebbing observed after both 24h and 48h treatment.

### The mechanisms of fucoxanthin and LY-294002 are different in apoptosis

The morphological differences observed above were further examined by fluorescence activated cell sorting (FACS) (S1G–S1L Fig). The apoptosis and necrosis levels of the treatments were measured using propidium iodide and annexin-V staining. Fucoxanthin did not increase the fractions of apoptotic cells after 24 h treatment (S1M & S1N vs S1O & S1P Fig), although these slightly decreased from 3.5% and 2.4% to 1.5 and 1.8% in the case of early and late apoptosis. The percentage of necrotic cells increased from less than 0.1% to 0.3%, which although statistically significant is probably not relevant (p = 0.011 in a one sided two sample t-test). Following a longer exposure for 48 h however, fucoxanthin increased the number of late apoptotic (from 2.2% to 6.7%) and necrotic cells (from 0.1% to 2.7%) compared to their respective fractions in vehicle-treated cultures (S1M and S1N Fig for control and Q, R for LY-294002, p = 0.056 and 0.013, respectively in a two-sample one sided t-test) (for more details of the results of FACS analysis, see the legend to S1 File). In turn, treatment with LY-294002 increased the fraction of early apoptotic cells from 3.5% and 3.3% to 6.8% and 6.7% and late apoptotic cells from 2.4% and 2.3% to 5.6% and 8.3% after 24 and 48 h treatments respectively, basically doubling the amount of apoptotic cells (two-sample one sided t-test p<0.05). The number of necrotic cells increased as well, but to a much lesser extent than in the case of fucoxanthin and not significantly after 48h (p = 0.08). The molecular mechanisms underlying these observations were further analyzed using transcriptomics, as shown in detail in S2 Fig.

### Compound effects are highly reproducible

Gene expression studies for U87MG cells treated with fucoxanthin and LY-294002 were performed at 24h and 48h time points, using the 72h IC_50_ concentrations of 20 μM and 200 μM respectively, to compare the modes of action of both compounds at early and late time points. To check the specificity and reproducibility of the drug responses as measured by gene expression analysis, a Principal Component Analysis (PCA) (S3 Fig) and a heatmap (S4 Fig) analysis were performed after normalizing the gene expression results following Robust Multi-array Average (RMA*)* [45] algorithm and the eBayes ANOVA (for details see Methods).

The results were highly reproducible. Both PCA and cluster analysis (S3 and S4 Figs) show a clear difference between the transcriptomic profiles exhibited by fucoxanthin and LY-294002 treatments. In both the cluster analysis and PCA the LY-294002 samples were more clearly different from control compared to fucoxanthin. This may indicate a more specific mechanism of action for LY-294002 compared to fucoxanthin.

### Differentially expressed genes after LY-294002 and fucoxanthin treatments

**Fig 3:**
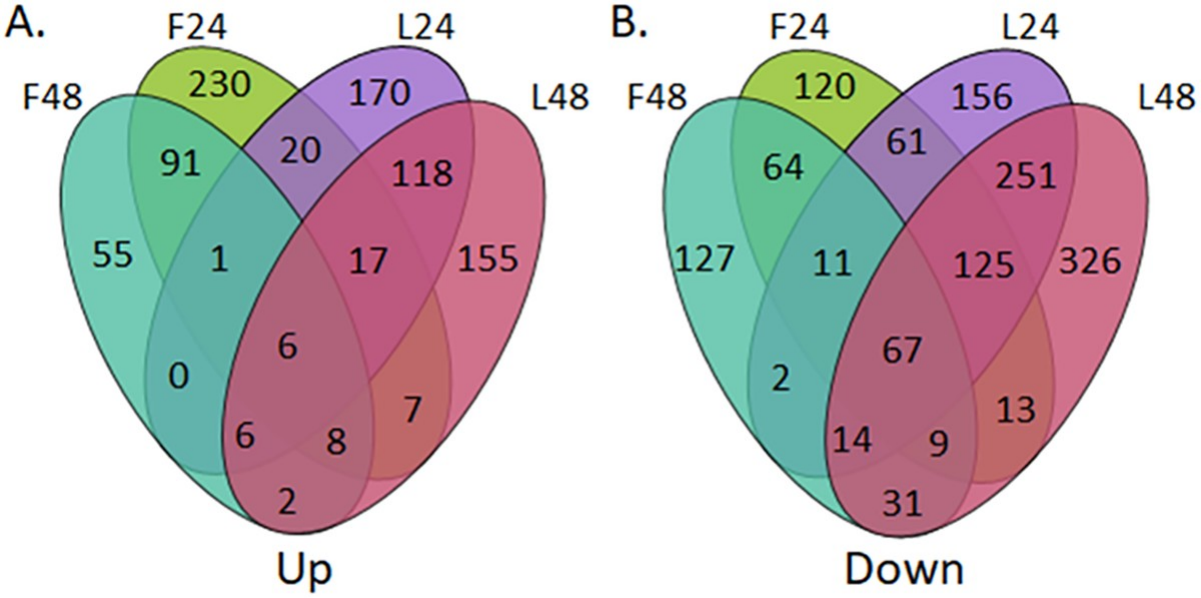
Venn diagram showing in-common and unique responses for the two treatments in gene expression space. Note that few up-regulated genes (A) are shared by the two treatments, in contrast to a much higher overlap in down-regulated genes (B). F24—represents the expression of a gene after 24h treatment with fucoxanthin; F48—represents the expression of a gene after 48h treatment with fucoxanthin; L24—represents the expression of a gene after 24h treatment with LY-294002; L48—represents the expression of a gene after 48h treatment with LY-294002.

Down-regulation of gene expression was the predominant cell response observed after both treatments. LY-294002 treatment showed a higher and more consistent down-regulation, with more genes shared between the 24h and 48h time-points, while fucoxanthin generated a less consistent response (Fig 3). Individual genes within these transcriptional profiles are annotated in S1 Table.

### Analysis of the 25 most differentially expressed genes from each treatment

After assembling overall patterns of differentially expressed genes (S1 Table, S5 Fig), we examined the 25 most differentially expressed genes in each condition to evaluate the consistency of the temporal response to each compound.

**Fig 4:**
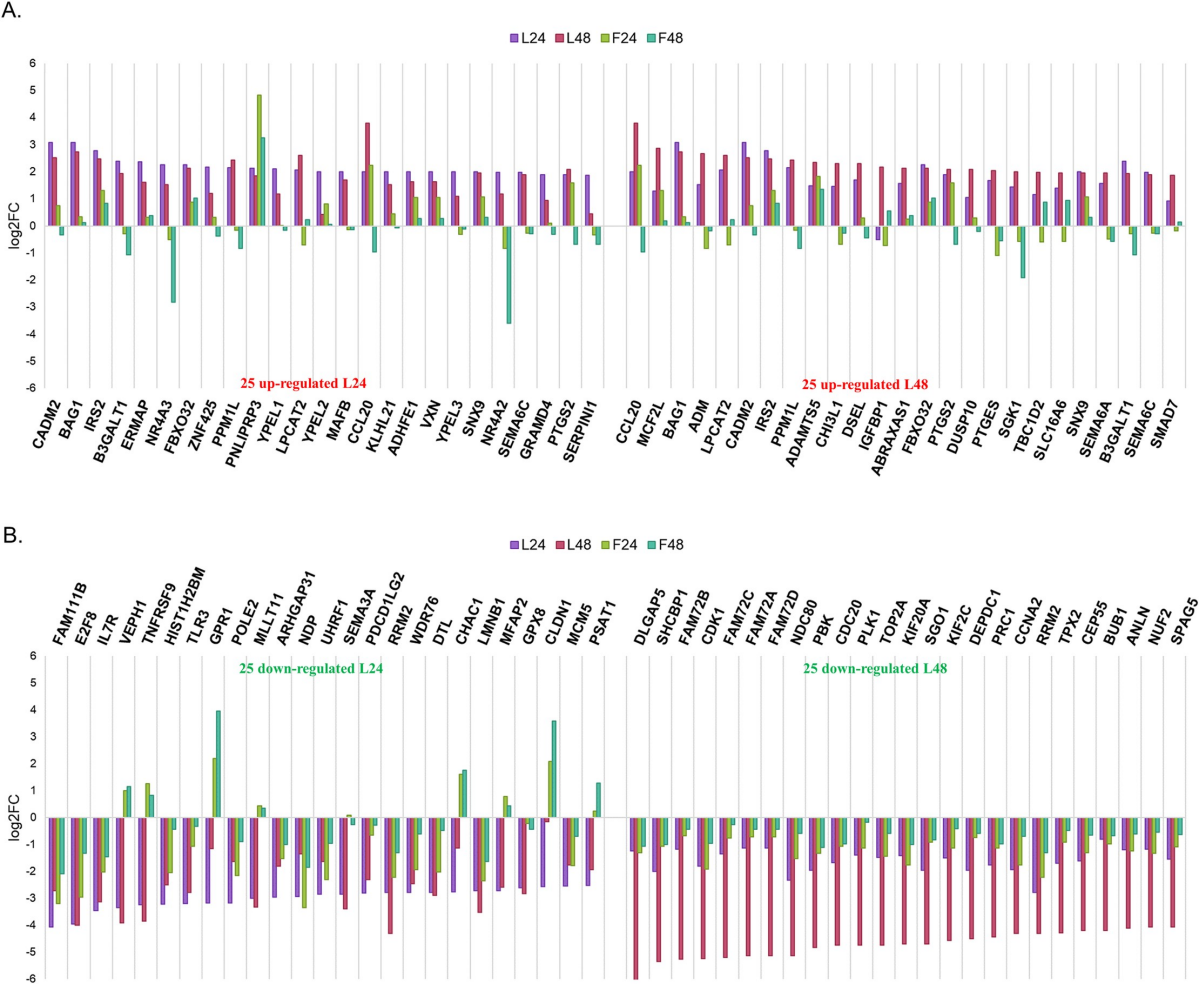
Gene expression signature difference between conditions using the 25 most differentially expressed up-regulated (A) and down-regulated (B) genes after LY-294002 at 24h (left side) and 48h (right side) treatment. The expression of an individual gene (log2FC) across all four conditions is marked in specific colors per condition (F24—light green, represents the expression of a gene after 24h treatment with fucoxanthin; F48—dark green, represents the expression of a gene after 48h treatment with fucoxanthin; L24—mauve represents the expression of a gene after 24h treatment with LY-294002; L48—pink, represents the expression of a gene after 48h treatment with LY-294002). The response to LY-294002 is consistent over time.

In the case of LY-294002 treatment at 24h (Fig 4A) and at 48h (Fig 4B), the 25 most differentially up- and down-regulated genes were mostly shared. The majority of genes show parallel responses at both time points, and with both drug treatments, although NR4A2, NR4A3, GPR1, CLDN1, CHAC1 and SGK1 show elevation only upon LY-294002 treatment, indicating a compound specific response in U87MG.

**Fig 5:**
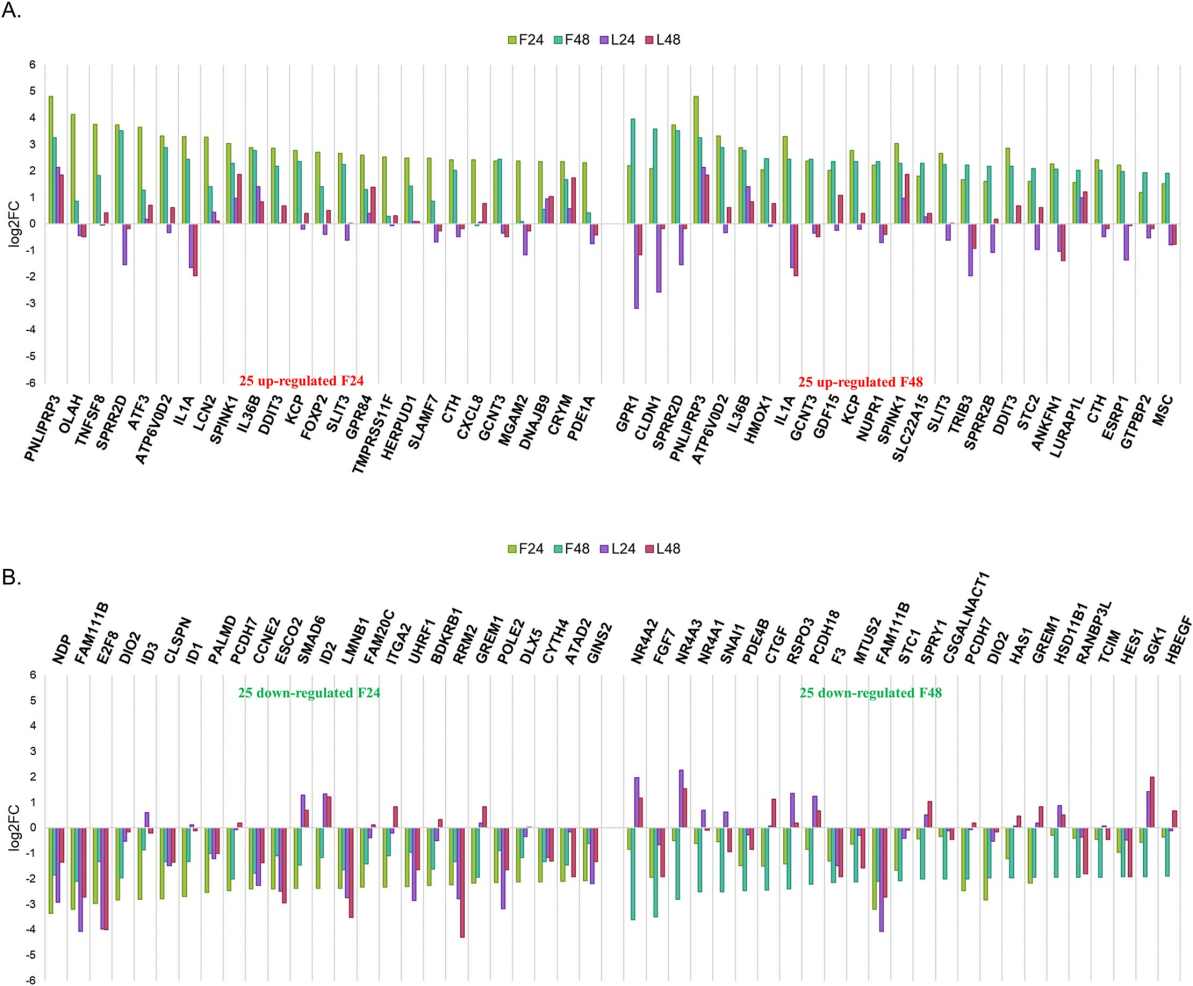
Gene expression differences between the 25 most differentially expressed up-regulated (A) and down-regulated (B) genes after fucoxanthin at 24h (left side) and 48h (right side) treatment. The expression of an individual gene (log2FC) across all four conditions is marked in specific colors per condition (F24—light green, represents the expression of a gene after fucoxanthin at 24h treatment; F48—dark green, represents the expression of a gene after fucoxanthin at 48h; L24—mauve represents the expression of a gene after LY-294002 at 24h; L48—pink, represents the expression of a gene after LY-294002 48h). The response to fucoxanthin is more variable at the two time points and differs from that of LY-294002.

Similarly, the 25 most differentially expressed genes in response to fucoxanthin at 24h and 48h were compared with those seen in LY-294002 treatments at the same time points (Fig 5). In this case, clear differences were seen between the gene expression profiles of the 25 most differentially expressed genes in each treatment.

Concentrating on differences between LY-294002 and fucoxanthin, amongst the up-regulated genes after 24h of LY-294002 treatment were two nuclear hormone receptors, NR4A2 and NR4A3, which were amongst the 25 most down-regulated genes after 48h of fucoxanthin treatment, illustrating a major difference in nuclear hormone induction between the two compounds. The NR4A2 and NR4A3 gene products regulate apoptosis in neutrophils [57], pointing to a potential difference in the molecular mechanisms underlying apoptosis in the two U87MG treatments used here.

### Pathways differentially modulated by fucoxanthin and LY-294002

Following analysis of individual gene expression changes, we next used WikiPathway analysis to obtain an overview of the main changes in signaling pathways produced by the two compounds; results for the 25 most differentially expressed pathways are shown in Table 1 (see S2 Table for entire list of expressed pathways with the corresponding up-regulated and down-regulated genes involved).

**Table 1:** The 25 most differentially expressed pathways sorted by the significance level (p-value) in U87MG cells between LY-294002 and fucoxanthin treatments for 24h (A) and 48h (B), using WikiPathway analysis. Pathways which are enriched in more than two cases are shown in bold. A gene is considered significantly differentially expressed if its FDR is < 0.1 and its absFC is > 1.

**A. 25 pathways most differentially expressed in response to 24h compound treatments**								
Pathways (LY-294002 24h)	Significance	Genes			Pathways (fucoxanthin 24h)	Significance	Genes	
	(-log P-value)	Up	Down			(-log P-value)	Up	Down
**Retinoblastoma Gene in Cancer**	**24.52**	**0**		**38**	**Retinoblastoma Gene in Cancer**	**31.71**	**0**	**42**
**DNA IR-damage and cellular response via ATR**	**14.08**	**1**		**27**	**DNA Replication**	**18.28**	**0**	**22**
Collagen biosynthesis and modifying enzymes	13.2	0		1	**Cell Cycle**	**12.96**	**1**	**28**
**DNA Replication**	**12.08**	**0**		**18**	**G1 to S cell cycle control**	**12.31**	**1**	**20**
G alpha (s) signaling events	11.66	1		0	**DNA IR-damage and cellular response via ATR**	**8.27**	**0**	**19**
**Cell Cycle**	**10.68**	**0**		**28**	O-linked glycosylation	8.21	1	0
Generic Transcription Pathway	9.6	0		1	Glycosaminoglycan metabolism	7.71	0	1
**G1 to S cell cycle control**	**9.3**	**1**		**18**	Histone Modifications	7.42	2	14
Glycosaminoglycan metabolism	9.09	1		0	Class A/1 (Rhodopsin-like receptors)	7.38	1	1
Collagen degradation	8.41	1		0	Photodynamic therapy-induced unfolded protein response	6.82	10	0
Histone Modifications	6.58	1		15	FBXL10 enhancement of MAP/ERK signaling in diffuse large B-cell lymphoma	6.6	0	11
**DNA Damage Response**	**6.49**	**0**		**16**	Olfactory receptor activity	6.18	1	0
FBXL10 enhancement of MAP/ERK signaling in diffuse large B-cell lymphoma	5.99	0		11	Gastric Cancer Network 1	5.45	0	9
Gastric Cancer Network 1	5.92	1		9	**DNA Damage Response**	**4.99**	**2**	**11**
miRNA Regulation of DNA Damage Response	5.53	0		18	Immunoregulatory interactions between a Lymphoid and a non-Lymphoid cell	4.88	1	0
Class A/1 (Rhodopsin-like receptors)	5.51	0		6	TCF dependent signaling in response to WNT	4.88	1	0
Deubiquitination	5.41	1		3	PI3K/Akt Signaling Pathway	4.75	10	24
Cell surface interactions at the vascular wall	5.4	0		1	Fc epsilon receptor (FCERI) signaling	4.38	1	0
Genotoxicity pathway	5.35	3		11	Deubiquitination	4.35	1	3
Signaling by ROBO receptors	5.23	1		0	Processing of Capped Intron-Containing Pre-mRNA	4.32	1	1
Neddylation	5.06	0		1	Signaling by the B Cell Receptor (BCR)	4.2	0	1
Regulation of Insulin-like Growth Factor (IGF) transport and uptake by Insulin-like Growth Factor Binding Proteins (IGFBPs)	4.57	0		1	Spinal Cord Injury	4.09	7	9
Nuclear Receptors Meta-Pathway	4.37	14		21	Lung fibrosis	3.87	4	7
Fc epsilon receptor (FCERI) signaling	4.34	2		0	miRNA Regulation of DNA Damage Response	3.86	2	12
Cilium Assembly	4.07	1		0	Cell surface interactions at the vascular wall	3.52	0	2

**B. 25 pathways most significantly enriched in response to 48h compound treatments**							
Pathways (LY-294002 48h)	Significance	Genes		Pathways (fucoxanthin 48h)	Significance	Genes	
	(-log P-value)	Up	Down		(-log P-value)	Up	Down
**Retinoblastoma Gene in Cancer**	**18.95**	**0**	**35**	**DNA Replication**	**7.03**	**0**	**10**
Collagen biosynthesis and modifying enzymes	15.89	0	1	Nuclear Receptors Meta-Pathway	6.95	11	16
Generic Transcription Pathway	11.53	0	1	Photodynamic therapy-induced unfolded protein response	6.53	8	0
Class I MHC mediated antigen processing & presentation	9.94	0	2	Hypertrophy Model	6.34	4	3
Glycosaminoglycan metabolism	9.78	2	0	**Retinoblastoma Gene in Cancer**	**6.23**	**1**	**12**
Collagen degradation	8.93	2	0	**G1 to S cell cycle control**	**5.18**	**1**	**9**
Deubiquitination	8.75	0	2	PI3K/Akt Signaling Pathway	**4.95**	**5**	**19**
Class A/1 (Rhodopsin-like receptors)	8.74	0	4	mRNA, protein, and metabolite induction pathway by cyclosporin A	4.82	4	0
**Cell Cycle**	**8.41**	**0**	**27**	NRF2 pathway	4.63	9	5
**DNA IR-damage and cellular response via ATR**	**8.34**	**1**	**22**	Genotoxicity pathway	4.39	4	5
Chromatin organization	7.45	0	1	O-linked glycosylation	4.18	1	1
TCF dependent signaling in response to WNT	7.11	1	0	SREBF and miR33 in cholesterol and lipid homeostasis	4.11	0	5
**DNA Replication**	**6.97**	**0**	**14**	Pancreatic adenocarcinoma pathway	3.97	5	5
Immunoregulatory interactions between a Lymphoid and a non-Lymphoid cell	6.93	0	1	Glycosaminoglycan metabolism	3.86	0	1
Processing of Capped Intron-Containing Pre-mRNA	6.45	1	1	Amino Acid metabolism	3.77	8	2
Fc epsilon receptor (FCERI) signaling	6.43	1	0	Spinal Cord Injury	3.61	4	7
Signaling by ROBO receptors	6.25	1	0	Photodynamic therapy-induced NFE2L2 (NRF2) survival signaling	3.57	4	1
Gastric Cancer Network 1	6.25	0	11	Photodynamic therapy-induced AP-1 survival signaling.	3.52	1	6
Neddylation	6.07	0	1	Target Of Rapamycin (TOR) Signaling	3.51	3	3
**G1 to S cell cycle control**	**5.85**	**0**	**16**	MAPK Signaling Pathway	3.13	9	7
**DNA Damage Response**	**4.89**	**0**	**15**	Extracellular vesicle-mediated signaling in recipient cells	3.01	1	4
HIV Life Cycle	4.76	0	1	EGF/EGFR Signaling Pathway	2.94	8	4
Regulation of Insulin-like Growth Factor (IGF) transport and uptake by Insulin-like Growth Factor Binding Proteins (IGFBPs)	4.51	1	1	Vitamin D Receptor Pathway	2.91	5	8
Fc gamma receptor (FCGR) dependent phagocytosis	4.26	0	1	ATF4 activates genes	2.87	5	0
Mitotic Prometaphase	4.1	0	1	Lung fibrosis	2.87	2	5

Many well-defined cellular signaling pathways are significantly modulated in common by the two compounds. Both compounds decreased the expression of genes from the “Retinoblastoma genes in cancer”, “DNA damage”, “DNA replication” and “Cell Cycle” pathways, suggesting a cytostatic effect. Interestingly the PI3K/Akt pathway, which we expected to be targeted by both inhibitors, is differentially affected only by fucoxanthin treatment, at both 24h (Table 1A) and 48h (Table 1B).

To obtain a different view on the pathway activity of the compounds we used GSEA (Gene Set Enrichment Analysis) with Hallmark and Gene Ontology classification. At all time points and treatment conditions, the Hallmark gene sets “E2F targets” and “G2M checkpoints” were down-regulated (FDR corrected p-value < 0.05, S3 Table). The GSEA results and WikiPathway approaches therefore reinforce each other, both analyses implicating the master regulator of the cell cycle E2F in co-ordinating the down-regulation of the genes in the G2M phase checkpoint, thereby producing cell cycle arrest.

Many other signaling pathways are affected by both treatments, listed in Table 1 and S2 Table. Amongst those of particular interest were the apoptosis and necrosis pathways, the cell cycle, and the EGFR pathways, elements of which have previously been seen in the U87MG growth response [58].

#### 1. Apoptosis pathway

From these pathway analyses, specific patterns of drug response can be inferred. One important pathway is apoptosis-related gene expression, summarized in Table 2A. A co-ordinated anti-apoptotic response to LY-294002 treatment is seen, with the same down-regulated apoptosis genes participating at both 24h and 48h treatment. Differential down-regulation of the transcripts for Caspase 1 and Caspase 10 is evident, together with down-regulation of the genes encoding FAS, BID, BIRC3, MAPK10 and HELLS. This suppression of the apoptosis pathway induced by LY-249002 may indicate the induction of a tumor cell survival program in U87MG cells, as suggested by the parallel up-regulation of growth-associated genes encoding the insulin-like growth factor 1 receptor (IGF1R), TNF receptor superfamily member 1B (TNFRSF1B) and the Bcl-2 like 11 (Bcl-2L11) genes.

**Table 2:** WikiPathway analysis. A, Apoptosis, B, PI3K/Akt, C, Retinoblastoma. L24, genes affected by LY-294002 at 24h; L48, genes affected by LY-294002 at 48h; F24, genes affected by fucoxanthin at 24h; L48, genes affected by fucoxanthin at 48h. All genes are listed in order of their level of expression.

**Differential expression of pathway genes**												
**A. Apoptosis**					**B. PI3K/Akt**				**C. Retinoblastoma**			
	**L24**	**L48**	**F24**	**F48**	**L24**	**L48**	**F24**	**F48**	**L24**	**L48**	**F24**	**F48**
**Down-regulated**	FAS	FAS	CASP2	TNFSF10	CCNE2	CCNE2	CCNE2	CCNE2	E2F1	E2F1	E2F1	CCNE2
	CASP1	CASP1	HELLS		GYS1	COL1A2	GYS1	ITGA2	SKP2	CDK2	SKP2	CCND3
	BIRC3	CASP10			COL1A2	TNC	ITGA5	PRLR	CDK2	CCNA2	CDK2	PLK4
	BID	BIRC3			COL6A3	PRLR	COL1A2	IL7R	CCNA2	SUV39H1	CCNA2	MCM6
	MAPK10	BID			PRLR	IL7R	LAMB1	ITGB8	MCM7	CCNE2	MCM7	MCM3
	BIRC5	MAPK10			IL7R	PIK3R3	ITGA2	ANGPT1	SUV39H1	CDC25A	SUV39H1	RFC3
	CASP8	BIRC5			ANGPT1	ANGPT1	PRLR	EFNA5	CCNE2	CCND3	CCNE2	MCM4
	HELLS	TNFSF10			HGF	FGF7	IL7R	FGF7	CDC25A	CDK1	CDC25A	POLE
		HELLS			EPHA2	HGF	LAMA5	HGF	CDK1	PLK4	CCND3	PRIM1
					PDGFRB	EPHA2	COL6A1	PDGFRA	PLK4	CCNB1	CDK1	BARD1
					CREB3L1	PDGFRB	ITGB8	LPAR6	CCNB1	CCNB2	PLK4	RRM2
					EIF4EBP1	PPP2R2B	CSF1	GNG2	CCNB2	MCM6	MCM6	CDK6
					TLR4	CREB3L1	ANGPT1	IL7	MCM6	RFC4	RFC4	
					TGFA	IL7	EFNA5	BCR	RFC4	TOP2A	TOP2A	
					CDK2	TLR4	FGF7	BDNF	TOP2A	CDC45	CDC45	
					CCND1	TGFA	HGF	TGFA	CDC45	MCM3	MCM3	
					BRCA1	CDK2	EPHA2	CDK6	MCM3	RFC3	RFC5	
						CCND3	GNG2	CCND3	RFC3	MCM4	RFC3	
						BRCA1	CREB3L1	SGK1	MCM4	PRIM1	MCM4	
							BCR		POLA1	ORC1	POLA1	
							TGFA		PRIM1	CHEK1	PRIM1	
							CDK2		ORC1	TTK	ORC1	
							CCND1		CHEK1	SMC2	CDT1	
							CCND3		BARD1	KIF4A	PCNA	
									TTK	RRM1	MSH6	
									SMC2	RRM2	BARD1	
									KIF4A	HMGB2	TTK	
									RRM1	TYMS	SMC2	
									RRM2	STMN1	KIF4A	
									TYMS	ANLN	RRM1	
									ANLN	RPA3	RRM2	
									CDC7	CDC7	TYMS	
									POLE2	POLE2	STMN1	
									FANCG	FANCG	ANLN	
									POLD3	POLD3	RPA3	
									H2AFZ		CDC7	
									CCND1		WEE1	
									E2F3		POLE2	
											CDC25B	
											SMC1A	
											POLD3	
											CCND1	
**Up-regulated**	**L24**	**L48**	**F24**	**F48**	**L24**	**L48**	**F24**	**F48**	**L24**	**L48**	**F24**	**F48**
	IGF1R	IGF1R	TRAF1	TP53	JAK2	CSF3	TSC1	FGF18				TP53
	TNFRSF1B		TNFRSF10B		IGF1R	JAK2	JAK2	NGF				
	BCL2L11				BCL2L11	ITGB3	IL2RB	ATF4				
					SGK1	ITGB8	SPP1	EIF4EBP1				
						KDR	KITLG	TP53				
						IGF1R	NGF					
						IL6	HSP90B1					
						SGK1	ATF4					
							C8orf44-SGK3					
							SGK3					

In sharp contrast, a different spectrum of down-regulated apoptotic genes accompanies growth inhibition by fucoxanthin. Here, the major down-regulated caspase is CASP2, accompanied by a much-restricted spectrum of other apoptotic genes. A full overview of changes observed in the apoptosis pathway is attached at S2 Fig, which also shows that it is the effector end of the pathway that is down-regulated most.

#### 2. PI3K/Akt pathway

Fucoxanthin has a transcriptomic effect on the PI3K/Akt pathway, increasing the expression of 10 of the genes involved in this pathway after 24h, 5 of which remain up-regulated after 48h treatment. At the same time, the expression of 24 genes is decreased at 24h, with 19 of these genes remaining down-regulated after 48h treatment (p<0.05 Benjamin Hochberg corrected Fisher exact test, S6 Fig).

Surprisingly, LY-294002 does not exert a significant transcriptional effect at the pathway level on the PI3K/Akt pathway (p>0.05 Benjamin Hochberg corrected Fisher exact test), even though it acts directly on this pathway by inhibition of PI3K. Only 4 genes from this large pathway at 24h, and 8 genes at 48h, were up-regulated, although 17 and 19 genes, respectively were down-regulated after 24h and 48h treatment (see S6 Fig for the wiring diagrams of the genes involved in the PI3K/Akt pathway). The reason behind this loss of statistical significance at the pathway level even though similar numbers of differentially expressed genes were involved in PI3K/Akt pathway, is that fucoxanthin has an effect on fewer genes that are differentially expressed between the two compounds, while LY-294002 has an effect on a higher number of differentially expressed genes (Table 2B).

Both compounds increased JAK2 expression, a component of the PI3K pathway (S6 Fig), LY-294002 at 24h and 48h, fucoxanthin only at the 24h time point. JAK2 modulates the PI3K/mTOR pathway [59] and has been described as a potential co-target for PI3K inhibitors [60]. Up-regulation of JAK2 could reflect a pro-proliferative role within the glioblastoma cells as they react to the growth inhibition caused by down-regulation of the PI3K pathway. Several other pro-proliferative genes (Bcl-2, SGK1, IGF1R) show similar induction patterns in the PI3K pathway itself (S6 Fig). The observation of SGK1 up-regulation is of particular interest since this kinase has recently been shown to be a key survival kinase for glioblastoma stem cells [61].

#### 3. Retinoblastoma pathway

Fucoxanthin has a marked transcriptomic effect on the Retinoblastoma pathway: 42 genes in this pathway are down-regulated at 24h, with 12 remaining down-regulated after 48h treatment (Table 2C and S7 Fig). LY-294002 also down-regulates gene expression within this pathway, with 35 genes showing down-regulation at 24h, 19 of which remain down-regulated at the 48h treatment time (S7 Fig).

Again, down-regulated genes appear mostly at the effector end of the pathway. Such genes include the cyclin dependent kinases CDK1 and CDK2, together with cyclins D4 and D6, and the main cell cycle initiator transcription factor E2F. *TP53* is the only gene up-regulated in the Retinoblastoma pathway, and this only in response to fucoxanthin at 48h. The results suggest that cell cycle arrest is effected by down-regulation of the retinoblastoma pathway by both compounds. However the lower amount of cell cycle arresting genes at 48h in the fucoxanthin treatment and the *TP53* gene up-regulation may indicate genotoxicity [62, 63].

### Fucoxanthin is synergistic in combination with LY-294002 on U87MG

The results of the transcriptomics strongly suggest that the two compounds fucoxanthin and LY-294002 are working in different ways.

**Fig 6:**
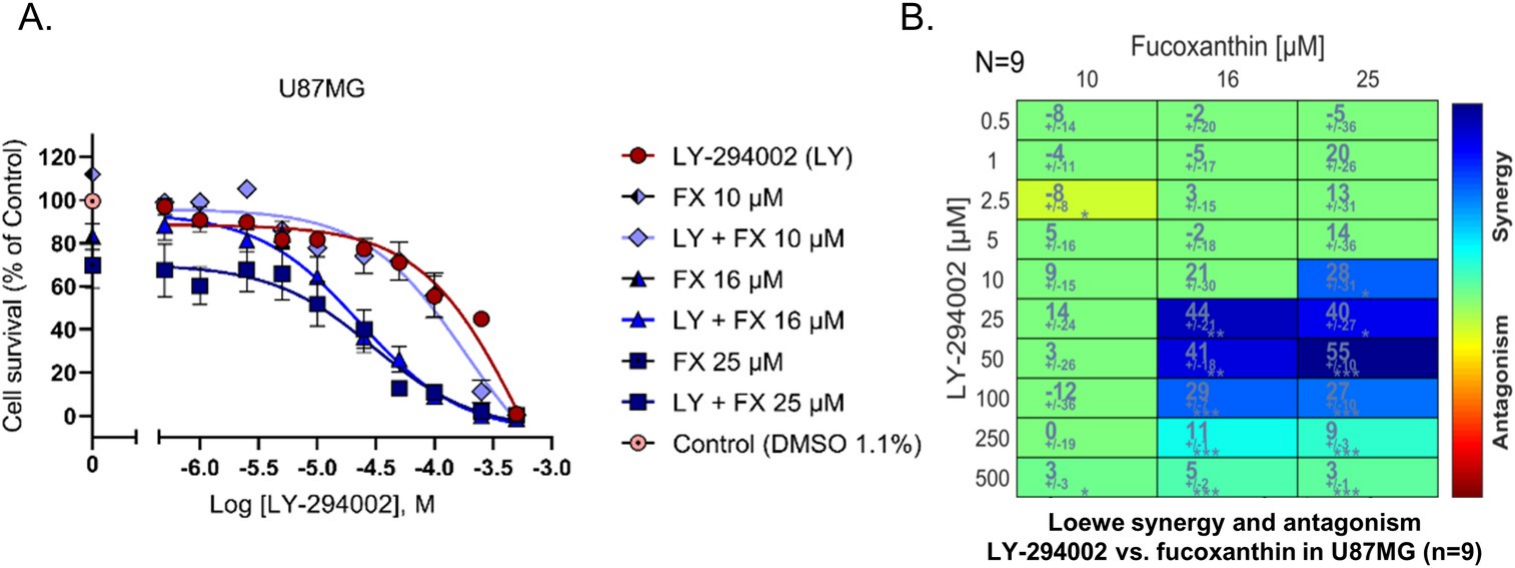
LY-294002 and fucoxanthin are synergistic. A) Dose response curves of LY-294002 and fucoxanthin using three concentrations of fucoxanthin (10μM, 16 μM and 25 μM) with 10 separate concentrations of LY-294002. B) Loewe synergy analysis.

To address the question whether fucoxanthin and LY-294002 when combined are synergistic in the U87MG system, we first analyzed the synergy of the two compounds in combination experiments based on Loewe synergy (Fig 6), observing significant synergy in the concentration range of 10–100μM LY-294002 combined with 16, 20 and 25μM fucoxanthin. We did not test combinations with fucoxanthin at concentrations higher than 25μM, because it causes potent inhibition of cell proliferation (see Fig 2A, 72h relationship). In other words, at 50 or 100μM fucoxanthin, there would be full suppression of proliferation and there is no point to examine the effect of LY-294002. When we tested the effect of the combination containing 10μM fucoxanthin (concentration lower than IC_50_), we found no synergy, so we did not attempt to test the effect of the combinations containing even lower concentrations of fucoxanthin. Therefore our range is: 10, 16 and 25μM.

**Fig 7:**
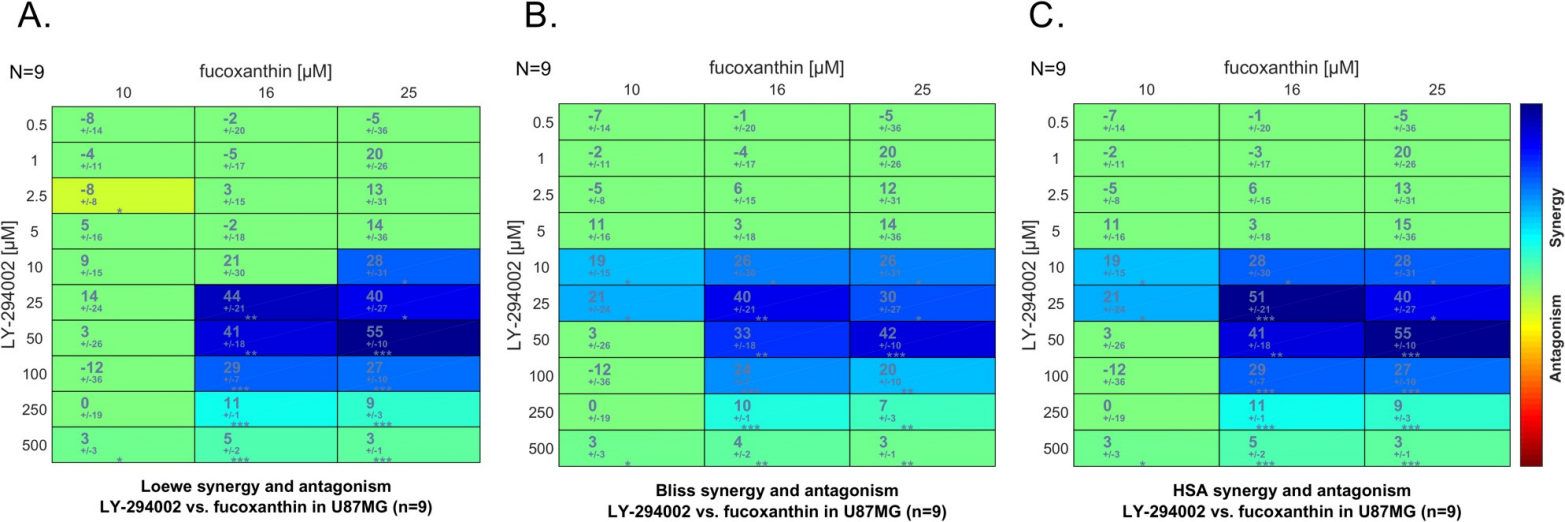
The synergy model used does not affect the synergy observed between fucoxanthin and LY-294002. A) Loewe synergy model, additive model, B) Bliss synergy model, product-based model, C) Highest Single Agent model–only one agent’s effect is considered. In all figures, the color indicates the degree of synergism and the values indicate synergy scores calculated as described in [43]. The higher the synergy is, the darker blue the background, and the higher the synergy score. Asterisks indicate significance of the synergy scores obtained following a one sample t-test (*p < 0.05; **p <0.001, ***p < 0.0001; the number of replicates (N) is shown on the left top corner of the matrix display).

In order to explore whether the observation of synergy was purely due to the particular synergy metric chosen, we next performed synergy analysis using the Bliss and Highest Single Agent (HSA) methods, implemented in the Combenefit package [43]. Combenefit provides a set of metrics (or scores) which captures information about the synergy distribution. As can be seen in Fig 7, while numerical synergies derived differ to an extent between methods, all methods identify synergy in the same concentration range of the compound combination used.

Fucoxanthin and LY-294002 treatments synergize, with maximal synergy being observed in these studies at 50 μM LY-294002 and 25 μM fucoxanthin, well below their individual IC_50_s (Fig 6). These data indicate that there are elements in the two response pathways that complement each other, thereby enhancing growth inhibition.

## Discussion

The feasibility of using gene expression as a measure of phenotypic change has been established for several systems, including the comparison of normal and glioblastoma-derived neural stem cells [64], drug screening [36] and toxicogenomics [65]. Here we use gene expression to gain insight into drug response in the glioblastoma cancer cell line U87MG, using two compounds with anti-proliferative effects, the prototypic pan-subtype specific PI3K inhibitor LY-294002 and the natural product fucoxanthin. Our report contains the first genome wide expression profile of changes associated with the exposure to fucoxanthin, a widely consumed natural carotenoid present in seaweeds, and illustrates the power of gene expression approaches to discover novel drug synergies in the nutri-pharmaceutical arena.

The U87MG cell system is widely used in glioblastoma research [66]. Although there has been much discussion about the origins and use of this cell line in studies of human glioblastoma [67, 68], the U87MG cell line remains one of the standard workhorses of drug discovery for this devastating disease.

In U87MG cells, fucoxanthin has previously been suggested to activate apoptosis *via* inhibition of the PI3K/Akt/mTOR pathway [30]. In the current study, we have used genome-wide gene expression to compare and contrast the effects of fucoxanthin on the PI3K and other pathways in U87MG cells, comparing its effects with those of a widely used non-selective PI3K inhibitor LY-294002.

We treated U87MG glioblastoma cells for 24h and 48h with each compound at its experimentally determined 72h anti-proliferative IC_50_ (Fig 2), reasoning that the “early” gene expression changes observed in response to drug treatment might be more reliable and informative than those accompanying later time points at which there could be substantial toxicity.

Indeed, the over-expression of TP53 at 48h of fucoxanthin treatment may indicate the potential toxicity of this natural product at this time point, since increased TP53 expression is a marker of toxicity [69, 70]. U87MG harbors wild-type TP53, which provides part of its defense against genomic damage [62, 63]. The possibility of emerging toxicity at 48h is also suggested by the FACS data (S1 Fig) which indicate increased necrosis. No pro-apoptotic genes are up-regulated by either fucoxanthin or LY-294002 at any time point tested (specifically illustrated in the apoptosis pathway analyses for U87MG shown in S2 Table).

U87MG cells respond to growth inhibition by both LY-294002 and fucoxanthin by activating new signaling pathways centered on growth and proliferation. These responses may represent survival pathways activated by growth inhibition, mirroring previous observations of PDGFR inhibitor resistance [71] and possibly reflecting the stemness of both the U87MG cell line and GBM tumors in general [72].

In the case of LY-294002, we observed the sustained up-regulation of JAK2 and insulin receptor substrate 2 at both 24h and 48h in the PI3K pathway, although for fucoxanthin, JAK2 was only observed as up-regulated at 24h. To escape PI3K inhibition, the glioblastoma cells may hence be inducing survival factors to activate alternative growth mechanisms through JAK2. The involvement of JAK2 in the cellular response to PI3K/Akt inhibition parallels other reports in which the JAK2 and PI3K pathways have been found to interact [28, 29].

Beyond the differences between the 2 treatments, many similarities can be seen: both fucoxanthin and LY-294002 arrest the cell cycle by down-regulating the cyclin dependent kinases CDK1 and CDK2, together with cyclins D4 and D6, and the main cell cycle initiator transcription factor E2F. Cell cycle regulation is being exerted through the retinoblastoma pathway, one of the most significantly down-regulated pathways in the case of both compounds at both time points. Close scrutiny shows that the effectors of the retinoblastoma pathway are also down-regulated, suggesting that the cells are entering a non-mitotic state.

Our results based on drug induced gene expression analysis support the orthogonal screening strategy being adopted within the WINDOW Consortium [73]. U87MG glioblastoma cells display a coordinated survival response upon LY-294002 treatment, involving both PI3K and JAK2. Combining inhibitors of these 2 targets has proven effective in other cancer cell systems such as in lung cancer cells [74], ovarian cancer cells [75] and also in murine xenografts models where combined PI3K/mTOR and JAK inhibitors showing potent efficacy of Philadelphia-like acute lymphoblastic leukemia [76] and should be tested in glioblastoma, along with other combinations suggested by the current data.

It is interesting to note that the gene expression profile observed for LY-294002 was more consistent than that of fucoxanthin. The disparity of gene expression responses between the 24h and 48h time points for fucoxanthin may indicate that this compound exhibits more complex drug-induced gene expression effects over time than does LY-294002. This hypothesis requires further investigation but is consistent with the observed metabolic lability of fucoxanthin in whole animal studies and the production of a new antiproliferative active metabolite, fucoxanthinol [77]. In parallel experiments not reported here, we have confirmed that fucoxanthinol inhibits U87MG cell proliferation, but do not yet know whether fucoxanthin is extensively metabolized to fucoxanthinol by glioblastoma cells themselves.

Although the effectiveness of fucoxanthin as a brain cancer treatment remains highly speculative [33], its active metabolite fucoxanthinol has shown encouraging effects in animal models of other cancers such as osteosarcoma [78], leukemia [79], colorectal cancer [80], and prostate cancer [81], supporting further studies of both fucoxanthin and fucoxanthinol in glioblastoma.

The remarkable divergence of gene induction and repression between the two anti-proliferative compounds in this cell system, reported for the first time, showing a combinatorial synergy between the synthetic compound and natural product, is predicted in part by the divergence in drug-induced gene expression signatures observed for the two compounds.

Detailed knowledge of drug response may thus be helpful in designing combination therapy approaches, especially where synergy between drugs and natural products can be demonstrated.

## Conclusions

Our studies illustrate the power of microarray-based transcriptomics to compare and contrast genome-wide gene expression changes accompanying drug response in human cancer cells, in this case using the U87MG human glioblastoma cell system. By coupling gene expression analysis to signaling pathway analysis, we have been able to clearly differentiate the effects of two target compounds, the prototypic PI3K inhibitor LY-294002 and the marine carotenoid fucoxanthin, previously thought to share common mechanisms of action.

Also, by focusing transcriptomics on a particular biological event (in our case, the induction of growth arrest in the U87MG glioblastoma cancer cell), we have been able to define and dissect important elements of the growth control pathways that can be induced in these cells. The remarkably wide divergence of gene induction and repression events accompanying treatment with the two anti-proliferative compounds in this model cell system, reported for the first time, raises the possibility of more widely exploiting the complementarity between the effects of synthetic compounds (such as kinase inhibitors) and natural products (such as marine carotenoids).

To this end, we have experimentally demonstrated a combinatorial synergy in growth arrest between the two anti-proliferative compounds used in this study, which can in part be predicted by the divergence in their drug-induced gene expression signatures. A detailed knowledge of genome-wide gene expression changes accompanying drug response may therefore be helpful in predicting and designing further novel nutri-pharmaceutical combinations to exploit such synergies.

## Supporting information

S1 Table DEGs per state. (XLSX)

S2 Table DEG pathway lists. (XLS)

S3 Table GSEA results. (XLSX)

S1 File FACS materials. (PDF)

S1 Fig Cell morphology and flow cytometry analysis after fucoxanthin and LY-294002 treatment. Representative images of cultured U87MG cells, analysis of flow cytometry charts and statistical analyses of the percentages of apoptotic and necrotic cells. Cells treated with vehicle for 24 h (A, G, M) and 48 h (B, H, N). Cells treated with 200 μM fucoxanthin for 24 h (C, I, O) and 48 h (D, J, P). Cells treated with 20 μM LY-294002 for 24 h (E, K, Q) and 48 h (F, L, R). Viable cells are shown in the lower left quarter (Q2-LL), early apoptotic cells are shown in the lower right quarter (Q2-LR), late apoptotic cells are shown in the upper right quarter (Q2-UR) and necrotic or mechanically damaged cells are shown in the upper left quarter (Q2-UL). More apoptotic cells are seen after LY-294002 compared to fucoxanthin treatment and more necrotic cells are seen after fucoxanthin compared to LY-294002 treatment. The stars compare time matched control and treated samples, using a one sided two sample t-test: *p <0.5 **p<0.01 ***p<0.001.
(TIF)

S2 Fig Apoptosis WikiPathway map representations. L24, Apoptosis affected by LY-294002 at 24h; L48, Apoptosis affected by LY-294002 at 48h. F24, Apoptosis affected by fucoxanthin at 24h. L48, Apoptosis affected by fucoxanthin 48h, showing down-regulated (left tables, in green) and up-regulated genes (left tables in red) in response to individual treatments.
(TIF)

S3 Fig Three views of the PCA plots of the gene expression data. The first 3 Principal Components (PCs) plotted contain 73.5% of the variance. Each of the 3 PCs are indicated with their representative variances on the axes of the graphs, together with what they represent in the analysis. Note that the samples cluster tightly with respect to treatment and time conditions emphasizing concordance within the analysis.
(TIF)

S4 Fig Euclidean distance based heatmap and clustering of the samples. The samples were clustered based on treatment first and then by time. LY-294002 24h (L24), LY-294002 48h (L48) treatments, fucoxanthin 24h (F24) and fucoxanthin 48h (F48) treatments, Control 24h (C24), Control 48h (C48). Up-regulated genes are shown in red; down-regulated genes are shown in blue. Only significantly differentially expressed genes with absolute fold change above 1 are shown. Genes which are “E2F targets” and “G2M checkpoints” according to the Broad dataset are shown. The colors used are the same as those used in the PCA analysis.
(TIF)

S5 Fig Volcano plot of the 4 treatments showing the top 25 down-regulated genes (left side, in blue) and the top 25 up-regulated genes (right side, in red) accompanied by their level of expression, expressed as logarithm-based 2 fold changes (Log2FC, < -1 or > 1) and corrected p-value as logarithm-base 10 false discovery rate (Log10FDR P-value, <0.05). **A.** top 25 differentially expressed genes in U87MG responding to LY-294002 at 24h treatment; **B.** top 25 differentially expressed genes in U87MG responding to LY-294002 at 48h treatment; **C.** top 25 differentially expressed genes in U87MG responding to fucoxanthin at 24h; **D.** top 25 differentially expressed genes in U87MG responding to fucoxanthin at 48h. The complete list of DEGs and their annotation is shown in S1 Table.
(TIF)

S6 Fig PI3K/Akt signaling Pathway WikiPathway map representations. L24, PI3K/Akt signaling pathway affected by LY-294002 at 24h; L48, PI3K/Akt signaling Pathway affected by LY-294002 at 48h. F24, PI3K/Akt signaling Pathway affected by fucoxanthin at 24h. L48, PI3K/Akt signaling Pathway affected by fucoxanthin 48h, showing down-regulated (left tables, in green) and up-regulated genes (left tables in red) in response to individual treatments.
(TIF)

S7 Fig Retinoblastoma gene in cancer, WikiPathway map representations. **L24,** Retinoblastoma gene in cancer pathway affected by LY-294002 at 24h; **L48,** Retinoblastoma gene in cancer pathway affected by LY-294002 at 48h; **F24,** Retinoblastoma gene in cancer pathway affected by fucoxanthin at 24h; **L48**, Retinoblastoma gene in cancer pathway affected by fucoxanthin at 48h; together with the down-regulated (left table in green) and up-regulated genes (left table in red) in response to each individual treatment. It can be seen that the effector end of the pathway is down-regulated the most.
(TIF)
