# Supplementary material for: Transcriptomics predicts compound synergy in drug and natural product treated glioblastoma cells
Source: PLoS One. 2020 Sep 18;15(9):e0239551. doi: 10.1371/journal.pone.0239551 (PMC7500592; doi:10.1371/journal.pone.0239551)
Supplement: S1 File — (PDF) [file pone.0239551.s004.pdf]

Control 24 h

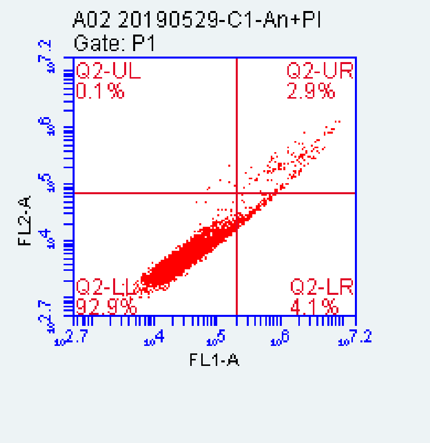

Plot 10: A02 20190529-C1-An+PI: Gated on P1	Count	Events / µL	% of This Plot	% of All	Mean FL1-A	Mean FL2-A	CV FL1-A	CV FL2-A
This Plot	5,544	370	100.00%	55.44%	112,695.74	15,398.77	403.27%	398.19%
Q2-UL	7	0	0.13%	0.07%	138,911.86	119,847.57	35.34%	34.45%
Q2-UR	161	11	2.90%	1.61%	2,084,101.39	286,701.50	81.44%	78.94%
Q2-LL	5,150	343	92.89%	51.50%	39,620.27	5,986.28	86.51%	67.31%
Q2-LR	226	15	4.08%	2.26%	372,690.77	33,378.63	46.10%	42.21%

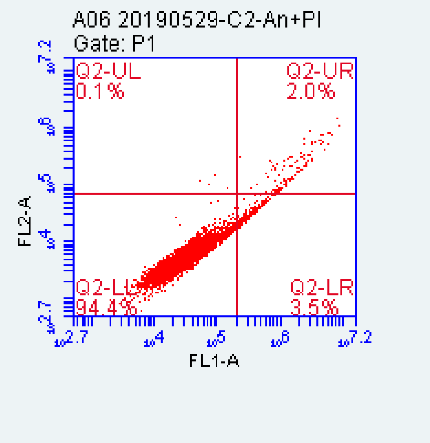

Plot 10: A06 20190529-C2-An+PI: Gated on P1	Count	Events / µL	% of This Plot	% of All	Mean FL1-A	Mean FL2-A	CV FL1-A	CV FL2-A
This Plot	5,387	414	100.00%	53.87%	90,444.71	11,957.47	432.46%	425.88%
Q2-UL	4	0	0.07%	0.04%	91,104.50	115,177.25	37.61%	19.89%
Q2-UR	109	8	2.02%	1.09%	2,163,329.88	268,847.33	78.88%	89.76%
Q2-LL	5,085	391	94.39%	50.85%	36,117.26	5,618.13	90.52%	66.24%
Q2-LR	189	15	3.51%	1.89%	356,624.15	32,177.82	43.62%	40.81%

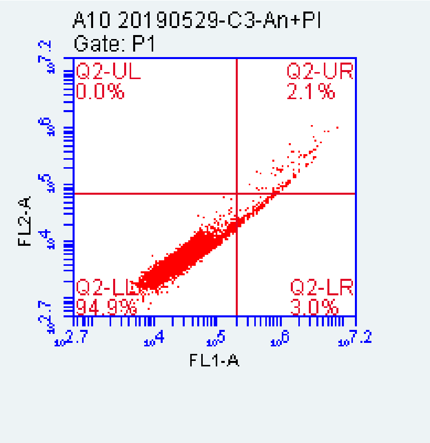

Plot 10: A10 20190529-C3-An+PI: Gated on P1	Count	Events / µL	% of This Plot	% of All	Mean FL1-A	Mean FL2-A	CV FL1-A	CV FL2-A
This Plot	4,848	346	100.00%	48.48%	83,032.75	11,200.59	400.95%	395.98%
Q2-UL	1	0	0.02%	0.01%	203,701.00	112,401.00	0.00%	0.00%
Q2-UR	100	7	2.06%	1.00%	1,861,181.74	238,090.19	73.43%	84.52%
Q2-LL	4,601	329	94.91%	46.01%	34,352.57	5,487.29	93.75%	70.60%
Q2-LR	146	10	3.01%	1.46%	398,388.63	35,150.64	42.47%	37.97%

Control 48 h

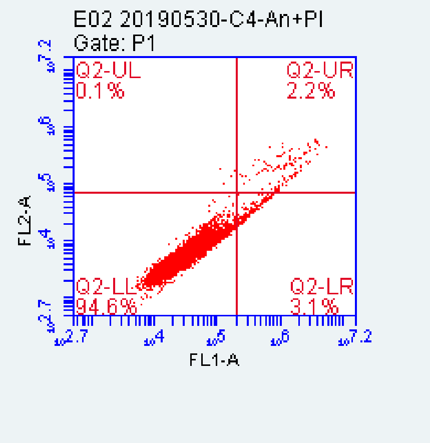

Plot 10: E02 20190530-C4-An+PI: Gated on P1	Count	Events / μL	% of This Plot	% of All	Mean FL1-A	Mean FL2-A	CV FL1-A	CV FL2-A
This Plot	5,436	388	100.00%	54.36%	83,092.31	12,294.19	338.42%	314.42%
Q2-UL	4	0	0.07%	0.04%	150,170.00	150,565.75	21.83%	21.51%
Q2-UR	122	9	2.24%	1.22%	1,548,149.89	224,409.11	67.25%	59.08%
Q2-LL	5,144	367	94.63%	51.44%	38,330.17	6,359.85	84.32%	67.35%
Q2-LR	166	12	3.05%	1.66%	391,833.86	36,963.60	40.65%	37.39%

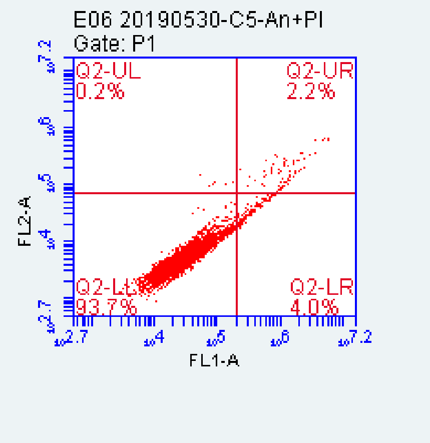

Plot 10: E06 20190530-C5-An+PI: Gated on P1	Count	Events / μL	% of This Plot	% of All	Mean FL1-A	Mean FL2-A	CV FL1-A	CV FL2-A
This Plot	3,411	341	100.00%	34.11%	83,804.22	11,443.71	352.83%	317.39%
Q2-UL	6	1	0.18%	0.06%	102,387.67	110,364.50	41.70%	14.05%
Q2-UR	74	7	2.17%	0.74%	1,546,582.53	203,771.45	79.93%	68.42%
Q2-LL	3,196	320	93.70%	31.96%	37,145.75	5,784.33	86.81%	70.58%
Q2-LR	135	14	3.96%	1.35%	385,755.07	35,603.62	45.75%	41.78%

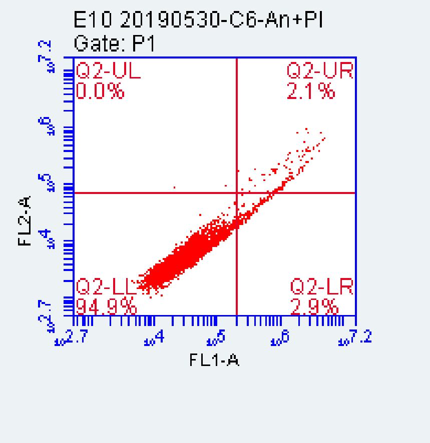

Plot 10: E10 20190530-C6-An+PI: Gated on P1	Count	Events / μL	% of This Plot	% of All	Mean FL1-A	Mean FL2-A	CV FL1-A	CV FL2-A
This Plot	5,122	394	100.00%	51.22%	82,344.75	11,414.42	358.27%	364.82%
Q2-UL	2	0	0.04%	0.02%	80,967.00	92,259.00	74.89%	3.31%
Q2-UR	110	8	2.15%	1.10%	1,686,027.24	225,678.62	64.21%	78.72%
Q2-LL	4,862	374	94.92%	48.62%	36,467.70	5,771.13	84.40%	65.98%
Q2-LR	148	11	2.89%	1.48%	397,560.23	36,460.94	43.33%	38.59%

FX 24 h

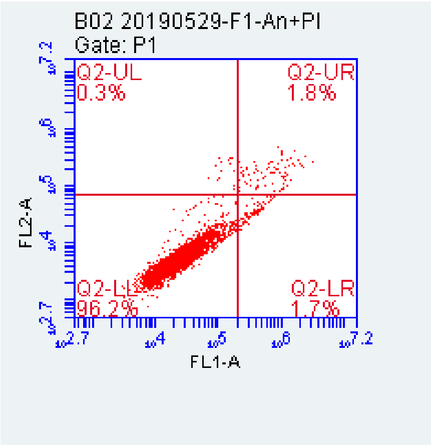

Plot 10: B02 20190529-F1-An+PI: Gated on P1	Count	Events / µL	% of This Plot	% of All	Mean FL1-A	Mean FL2-A	CV FL1-A	CV FL2-A
This Plot	4,837	372	100.00%	48.37%	48,732.52	9,960.74	308.27%	298.72%
Q2-UL	15	1	0.31%	0.15%	133,420.80	200,029.53	27.37%	40.70%
Q2-UR	88	7	1.82%	0.88%	872,645.22	185,753.84	70.02%	47.03%
Q2-LL	4,653	358	96.20%	46.53%	26,853.29	5,565.39	86.74%	79.14%
Q2-LR	81	6	1.67%	0.81%	394,774.75	36,265.93	39.76%	35.98%

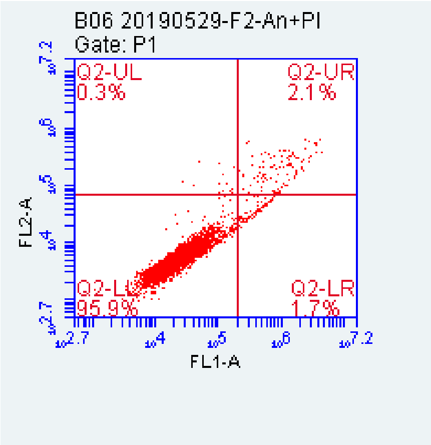

Plot 10: B06 20190529-F2-An+PI: Gated on P1	Count	Events / µL	% of This Plot	% of All	Mean FL1-A	Mean FL2-A	CV FL1-A	CV FL2-A
This Plot	4,711	337	100.00%	47.11%	59,973.26	11,383.28	401.19%	367.51%
Q2-UL	13	1	0.28%	0.13%	114,942.85	132,211.23	42.47%	47.61%
Q2-UR	101	7	2.14%	1.01%	1,301,739.23	244,258.71	74.93%	60.79%
Q2-LL	4,518	323	95.90%	45.18%	25,541.01	5,350.03	87.99%	68.37%
Q2-LR	79	6	1.68%	0.79%	432,529.42	38,814.06	38.74%	35.17%

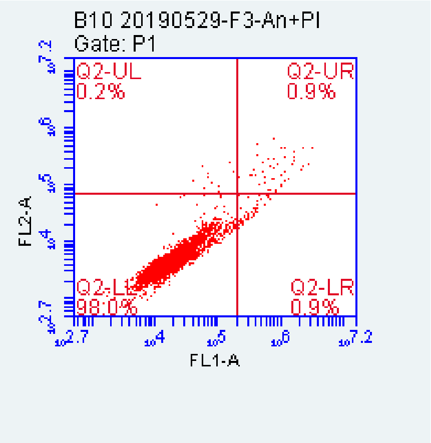

Plot 10: B10 20190529-F3-An+PI: Gated on P1	Count	Events / µL	% of This Plot	% of All	Mean FL1-A	Mean FL2-A	CV FL1-A	CV FL2-A
This Plot	5,000	294	100.00%	50.00%	34,819.91	7,675.96	354.93%	333.51%
Q2-UL	10	1	0.20%	0.10%	92,987.00	142,611.60	46.26%	37.62%
Q2-UR	45	3	0.90%	0.45%	1,039,591.40	221,621.09	68.13%	62.25%
Q2-LL	4,900	288	98.00%	49.00%	22,437.94	5,176.41	87.47%	75.23%
Q2-LR	45	3	0.90%	0.45%	365,381.31	35,918.80	35.39%	39.00%

FX 48 h

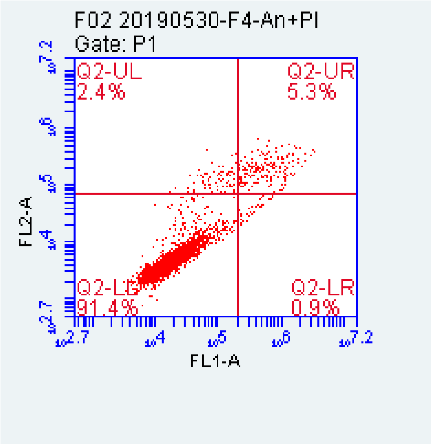

Plot 10: F02 20190530-F4-An+PI: Gated on P1	Count	Events / µL	% of This Plot	% of All	Mean FL1-A	Mean FL2-A	CV FL1-A	CV FL2-A
This Plot	3,438	191	100.00%	34.38%	66,374.58	20,499.59	329.31%	281.80%
Q2-UL	82	5	2.39%	0.82%	111,827.49	141,590.73	46.74%	44.31%
Q2-UR	182	10	5.29%	1.82%	754,097.94	218,098.55	79.95%	48.60%
Q2-LL	3,143	175	91.42%	31.43%	22,064.04	5,705.44	88.32%	93.91%
Q2-LR	31	2	0.90%	0.31%	401,058.94	40,031.94	39.86%	36.40%

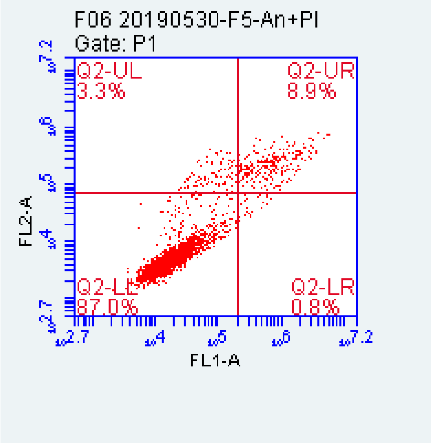

Plot 10: F06 20190530-F5-An+PI: Gated on P1	Count	Events / µL	% of This Plot	% of All	Mean FL1-A	Mean FL2-A	CV FL1-A	CV FL2-A
This Plot	2,903	132	100.00%	29.03%	112,940.88	34,138.73	340.44%	256.57%
Q2-UL	97	4	3.34%	0.97%	97,934.66	171,644.94	52.24%	53.53%
Q2-UR	257	12	8.85%	2.57%	984,818.68	257,249.94	91.69%	52.67%
Q2-LL	2,525	115	86.98%	25.25%	22,321.42	6,052.23	92.31%	108.05%
Q2-LR	24	1	0.83%	0.24%	371,154.96	44,168.96	40.62%	33.64%

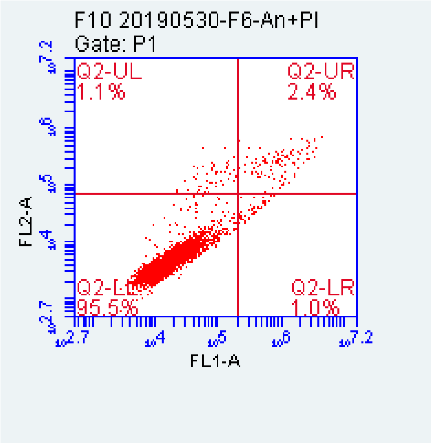

Plot 10: F10 20190530-F6-An+PI: Gated on P1	Count	Events / µL	% of This Plot	% of All	Mean FL1-A	Mean FL2-A	CV FL1-A	CV FL2-A
This Plot	4,740	135	100.00%	47.40%	52,950.78	13,775.06	439.58%	365.80%
Q2-UL	52	1	1.10%	0.52%	94,132.12	164,787.37	52.58%	49.38%
Q2-UR	112	3	2.36%	1.12%	1,211,824.23	279,918.40	75.32%	49.02%
Q2-LL	4,528	129	95.53%	45.28%	20,260.47	5,196.69	86.52%	86.42%
Q2-LR	48	1	1.01%	0.48%	388,086.04	38,403.67	41.33%	37.23%

LY 24 h

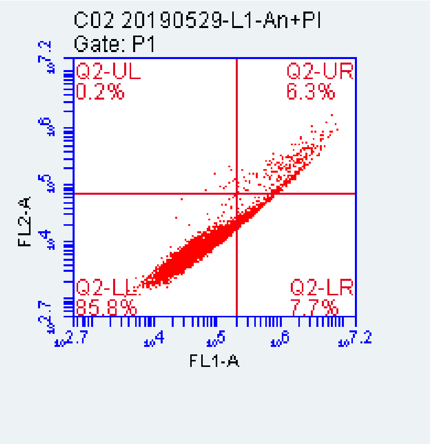

Plot 10: C02 20190529-L1-An+PI: Gated on P1	Count	Events / µL	% of This Plot	% of All	Mean FL1-A	Mean FL2-A	CV FL1-A	CV FL2-A
This Plot	4,871	256	100.00%	48.71%	192,378.54	25,098.57	290.46%	319.32%
Q2-UL	10	1	0.21%	0.10%	140,289.00	128,129.70	28.22%	34.32%
Q2-UR	307	16	6.30%	3.07%	1,845,026.76	248,600.63	74.12%	87.02%
Q2-LL	4,181	220	85.83%	41.81%	54,777.07	7,621.76	74.59%	61.32%
Q2-LR	373	20	7.66%	3.73%	375,943.21	34,281.05	42.51%	37.32%

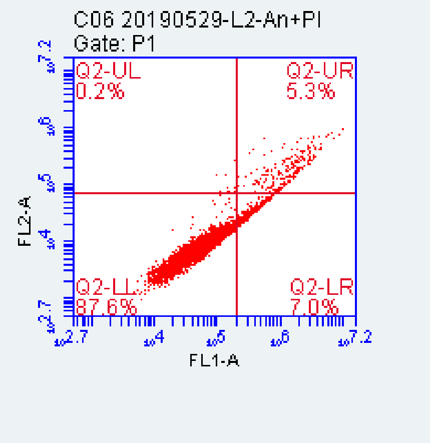

Plot 10: C06 20190529-L2-An+PI: Gated on P1	Count	Events / µL	% of This Plot	% of All	Mean FL1-A	Mean FL2-A	CV FL1-A	CV FL2-A
This Plot	4,905	307	100.00%	49.05%	170,954.37	21,434.65	307.88%	298.74%
Q2-UL	10	1	0.20%	0.10%	158,159.40	130,556.40	22.46%	53.44%
Q2-UR	258	16	5.26%	2.58%	1,863,671.18	235,081.90	76.90%	70.58%
Q2-LL	4,295	268	87.56%	42.95%	53,101.89	7,309.01	75.87%	62.47%
Q2-LR	342	21	6.97%	3.42%	374,414.71	34,468.11	42.94%	38.38%

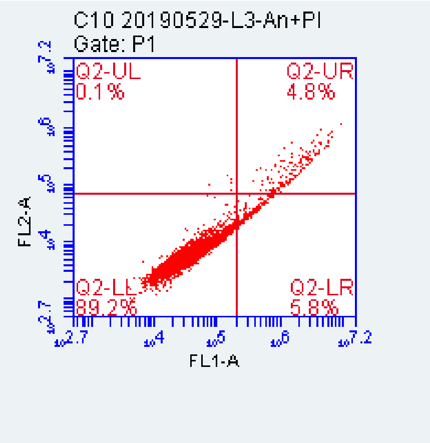

Plot 10: C10 20190529-L3-An+PI: Gated on P1	Count	Events / µL	% of This Plot	% of All	Mean FL1-A	Mean FL2-A	CV FL1-A	CV FL2-A
This Plot	3,392	200	100.00%	33.92%	168,975.17	20,456.76	347.94%	330.34%
Q2-UL	5	0	0.15%	0.05%	145,314.60	114,864.60	25.48%	20.28%
Q2-UR	163	10	4.81%	1.63%	2,217,056.56	255,312.59	72.93%	73.61%
Q2-LL	3,026	178	89.21%	30.26%	45,762.01	6,783.65	85.74%	67.57%
Q2-LR	198	12	5.84%	1.98%	366,571.42	33,695.70	40.19%	37.24%

LY 48 h

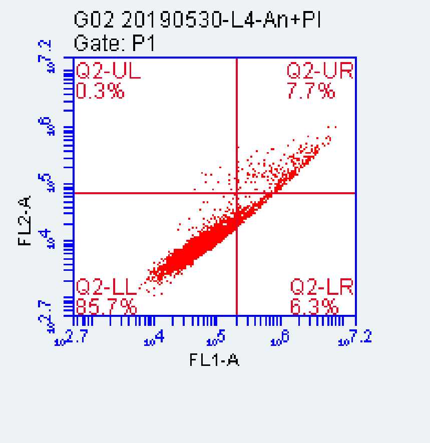

Plot 10: G02 20190530-L4-An+PI: Gated on P1	Count	Events / µL	% of This Plot	% of All	Mean FL1-A	Mean FL2-A	CV FL1-A	CV FL2-A
This Plot	4,010	211	100.00%	40.10%	206,320.25	27,088.95	277.68%	255.47%
Q2-UL	14	1	0.35%	0.14%	108,413.93	127,257.00	45.21%	26.37%
Q2-UR	307	16	7.66%	3.07%	1,729,520.31	219,111.94	74.61%	65.98%
Q2-LL	3,438	181	85.74%	34.38%	59,066.38	8,851.26	70.74%	61.94%
Q2-LR	251	13	6.26%	2.51%	365,710.99	36,442.69	40.87%	35.55%

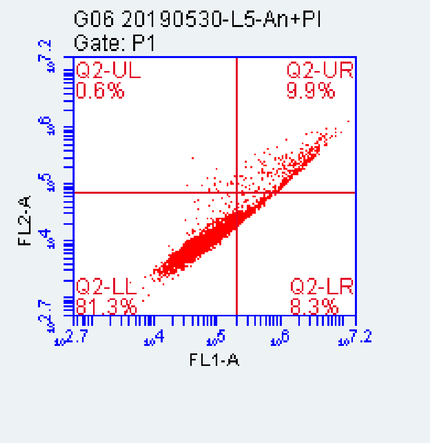

Plot 10: G06 20190530-L5-An+PI: Gated on P1	Count	Events / µL	% of This Plot	% of All	Mean FL1-A	Mean FL2-A	CV FL1-A	CV FL2-A
This Plot	3,779	172	100.00%	37.79%	299,536.82	36,827.06	267.82%	247.75%
Q2-UL	21	1	0.56%	0.21%	107,934.29	111,179.38	46.69%	41.36%
Q2-UR	375	17	9.92%	3.75%	2,159,953.41	254,939.85	73.86%	67.36%
Q2-LL	3,071	140	81.26%	30.71%	67,978.59	9,807.01	67.22%	60.99%
Q2-LR	312	14	8.26%	3.12%	355,571.23	35,624.72	40.86%	34.66%

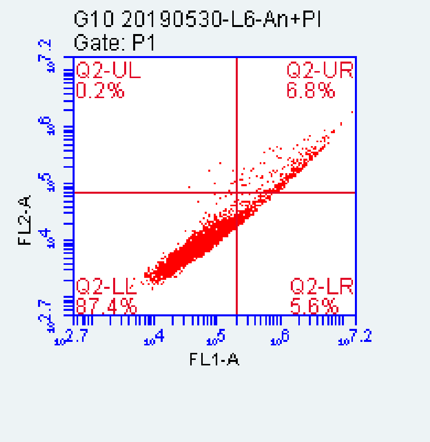

Plot 10: G10 20190530-L6-An+PI: Gated on P1	Count	Events / µL	% of This Plot	% of All	Mean FL1-A	Mean FL2-A	CV FL1-A	CV FL2-A
This Plot	4,027	201	100.00%	40.27%	218,263.70	26,657.19	330.94%	304.67%
Q2-UL	9	0	0.22%	0.09%	132,695.11	129,730.56	39.62%	36.98%
Q2-UR	273	14	6.78%	2.73%	2,232,782.72	250,752.18	80.58%	81.48%
Q2-LL	3,519	176	87.39%	35.19%	53,601.17	8,417.16	75.42%	67.27%
Q2-LR	226	11	5.61%	2.26%	352,130.48	35,865.49	38.69%	36.18%
