# Supplementary figures and images for: Transcriptomics predicts compound synergy in drug and natural product treated glioblastoma cells
Source: PLoS One. 2020 Sep 18;15(9):e0239551. doi: 10.1371/journal.pone.0239551 (PMC7500592; doi:10.1371/journal.pone.0239551)

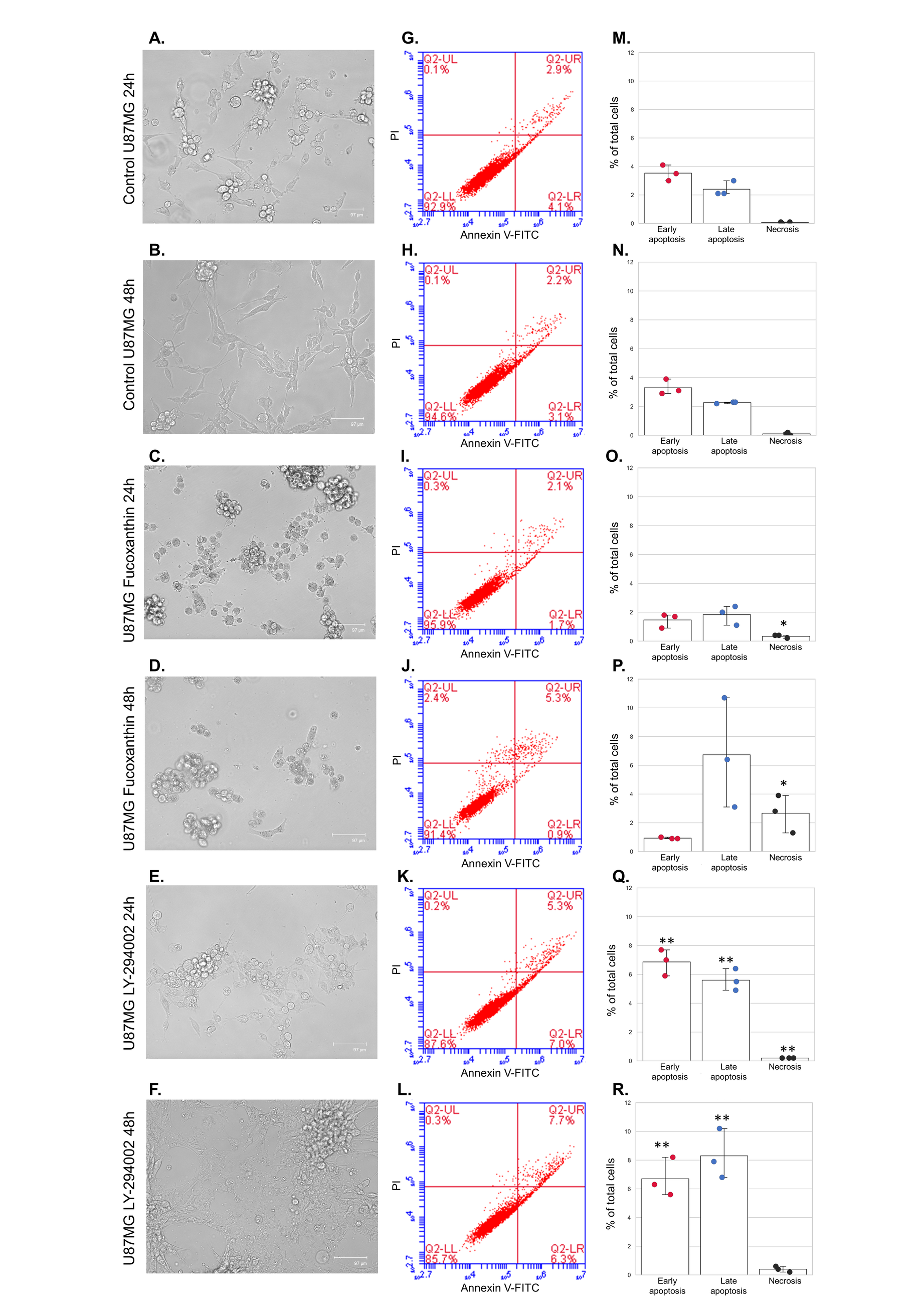

Supplement: S1 Fig — Representative images of cultured U87MG cells, analysis of flow cytometry charts and statistical analyses of the percentages of apoptotic and necrotic cells. Cells treated with vehicle for 24 h (A, G, M) and 48 h (B, H, N). Cells treated with 200 μM fucoxanthin for 24 h (C, I, O) and 48 h (D, J, P). Cells treated with 20 μM LY-294002 for 24 h (E, K, Q) and 48 h (F, L, R). Viable cells are shown in the lower left quarter (Q2-LL), early apoptotic cells are shown in the lower right quarter (Q2-LR), late apoptotic cells are shown in the upper right quarter (Q2-UR) and necrotic or mechanically damaged cells are shown in the upper left quarter (Q2-UL). More apoptotic cells are seen after LY-294002 compared to fucoxanthin treatment and more necrotic cells are seen after fucoxanthin compared to LY-294002 treatment. The stars compare time matched control and treated samples, using a one sided two sample t-test: *p <0.5 **p<0.01 ***p<0.001. (TIF) [file pone.0239551.s005.tif]

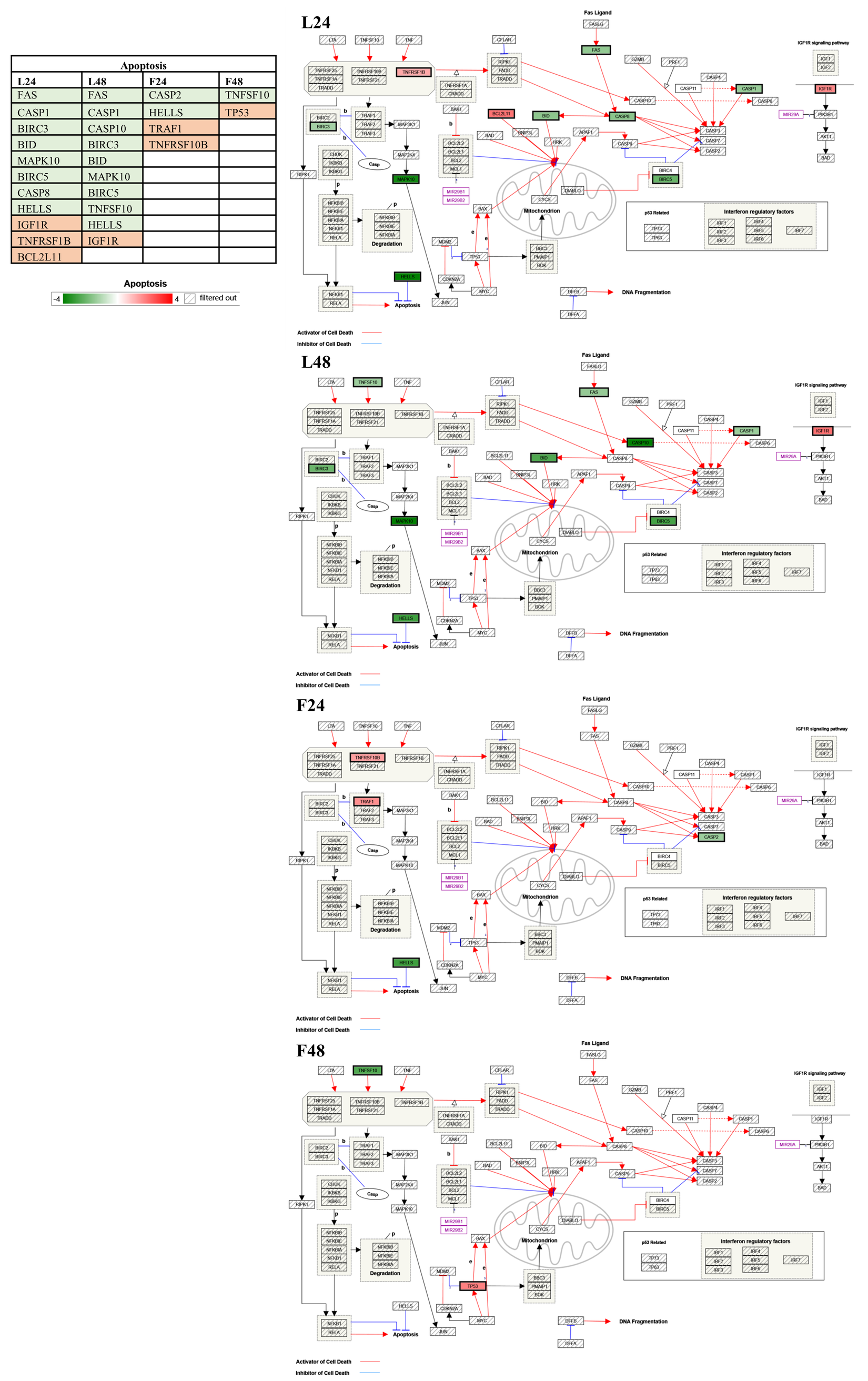

Supplement: S2 Fig — L24, Apoptosis affected by LY-294002 at 24h; L48, Apoptosis affected by LY-294002 at 48h. F24, Apoptosis affected by fucoxanthin at 24h. L48, Apoptosis affected by fucoxanthin 48h, showing down-regulated (left tables, in green) and up-regulated genes (left tables in red) in response to individual treatments. (TIF) [file pone.0239551.s006.tif]

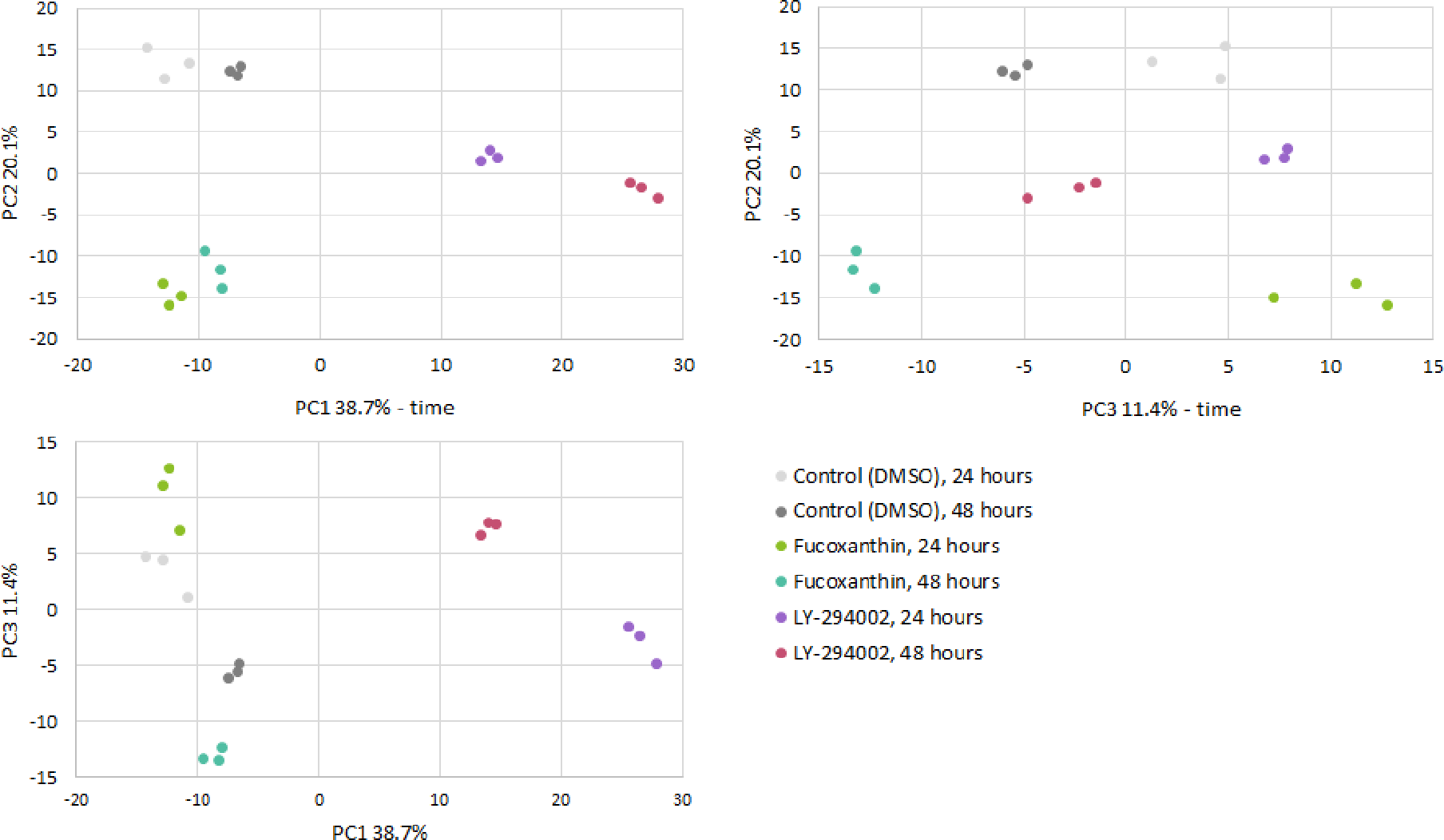

Supplement: S3 Fig — The first 3 Principal Components (PCs) plotted contain 73.5% of the variance. Each of the 3 PCs are indicated with their representative variances on the axes of the graphs, together with what they represent in the analysis. Note that the samples cluster tightly with respect to treatment and time conditions emphasizing concordance within the analysis. (TIF) [file pone.0239551.s007.tif]

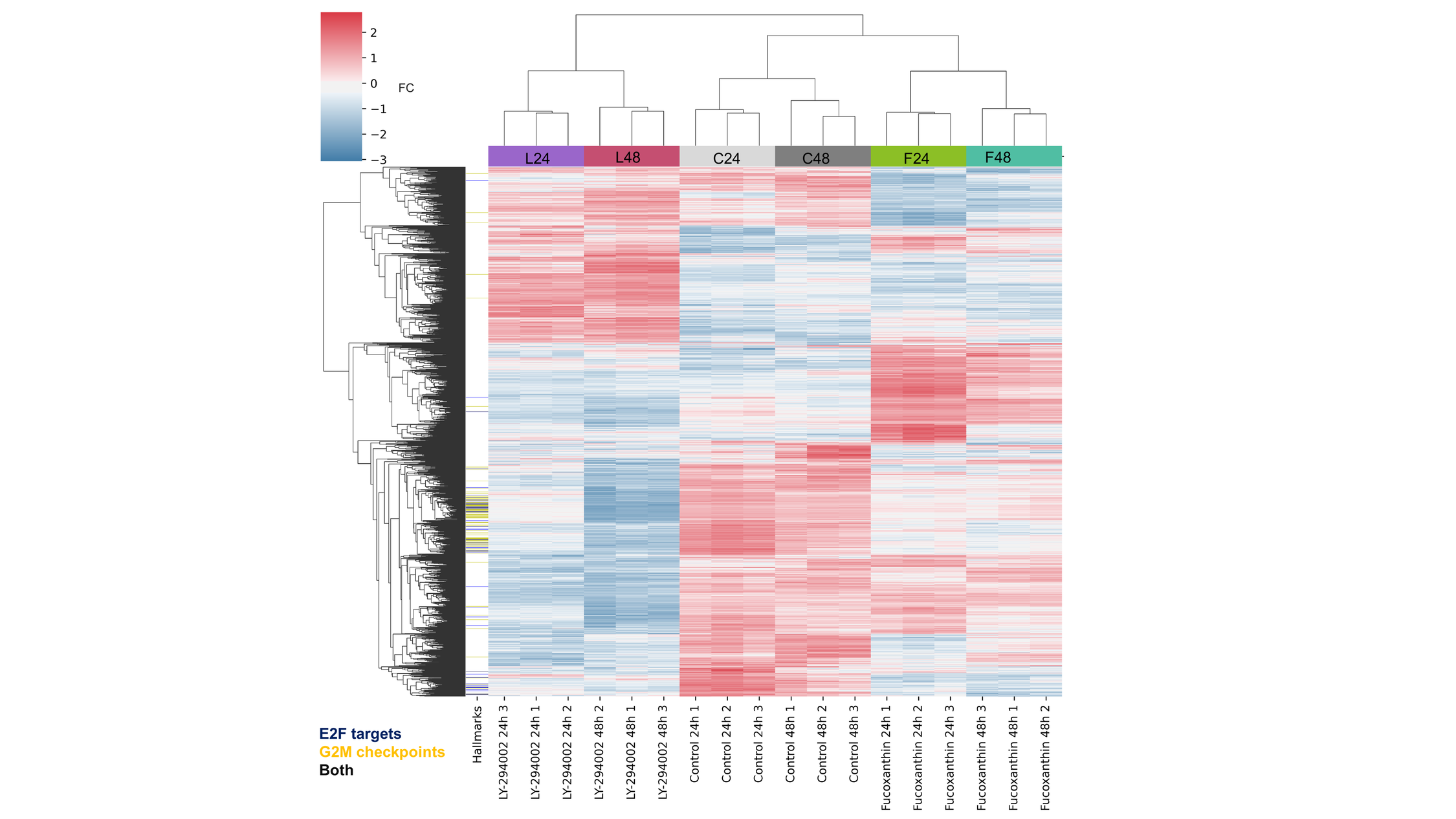

Supplement: S4 Fig — The samples were clustered based on treatment first and then by time. LY-294002 24h (L24), LY-294002 48h (L48) treatments, fucoxanthin 24h (F24) and fucoxanthin 48h (F48) treatments, Control 24h (C24), Control 48h (C48). Up-regulated genes are shown in red; down-regulated genes are shown in blue. Only significantly differentially expressed genes with absolute fold change above 1 are shown. Genes which are “E2F targets” and “G2M checkpoints” according to the Broad dataset are shown. The colors used are the same as those used in the PCA analysis. (TIF) [file pone.0239551.s008.tif]

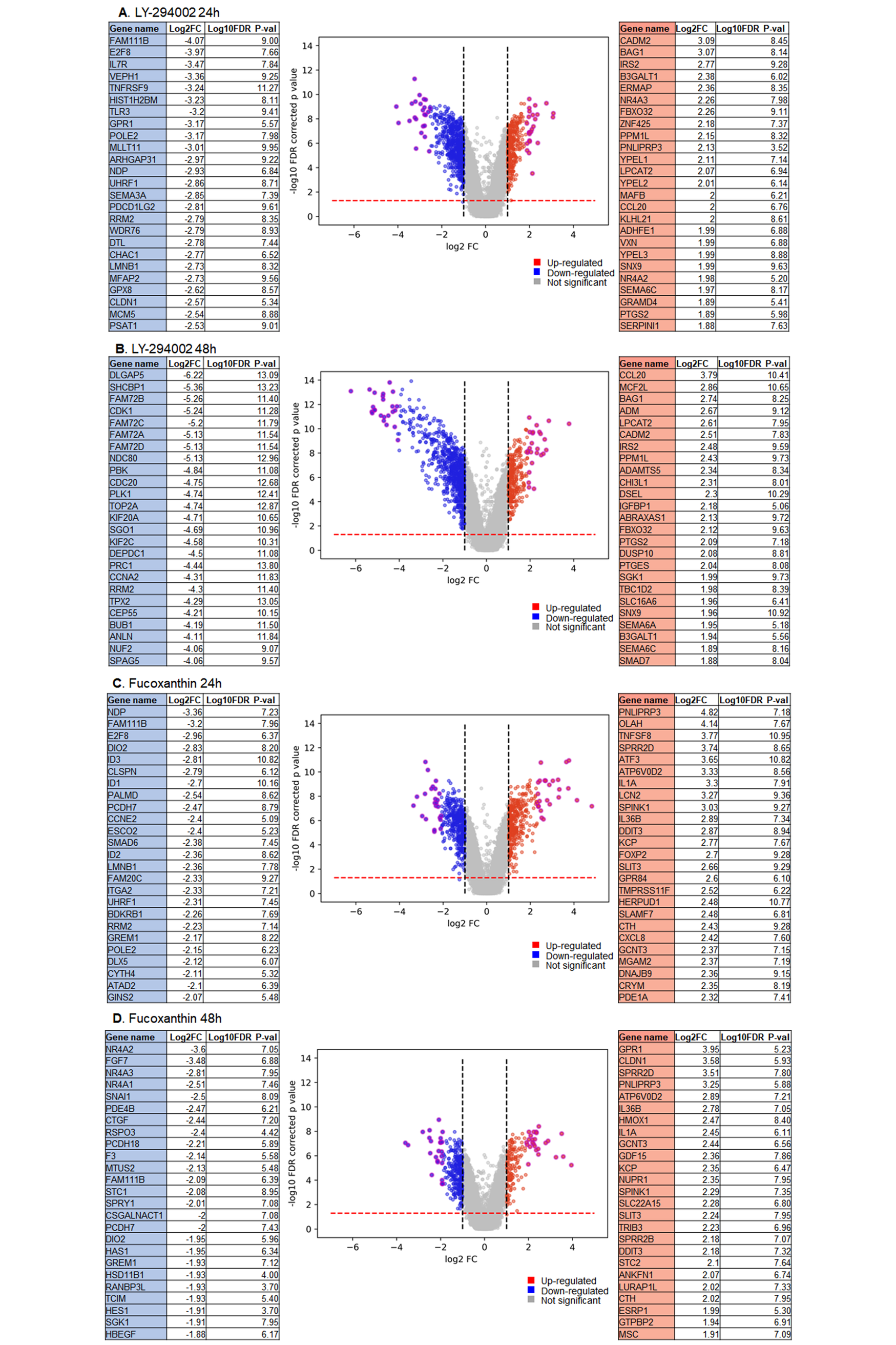

Supplement: S5 Fig — A. top 25 differentially expressed genes in U87MG responding to LY-294002 at 24h treatment; B. top 25 differentially expressed genes in U87MG responding to LY-294002 at 48h treatment; C. top 25 differentially expressed genes in U87MG responding to fucoxanthin at 24h; D. top 25 differentially expressed genes in U87MG responding to fucoxanthin at 48h. The complete list of DEGs and their annotation is shown in S1 Table. (TIF) [file pone.0239551.s009.tif]

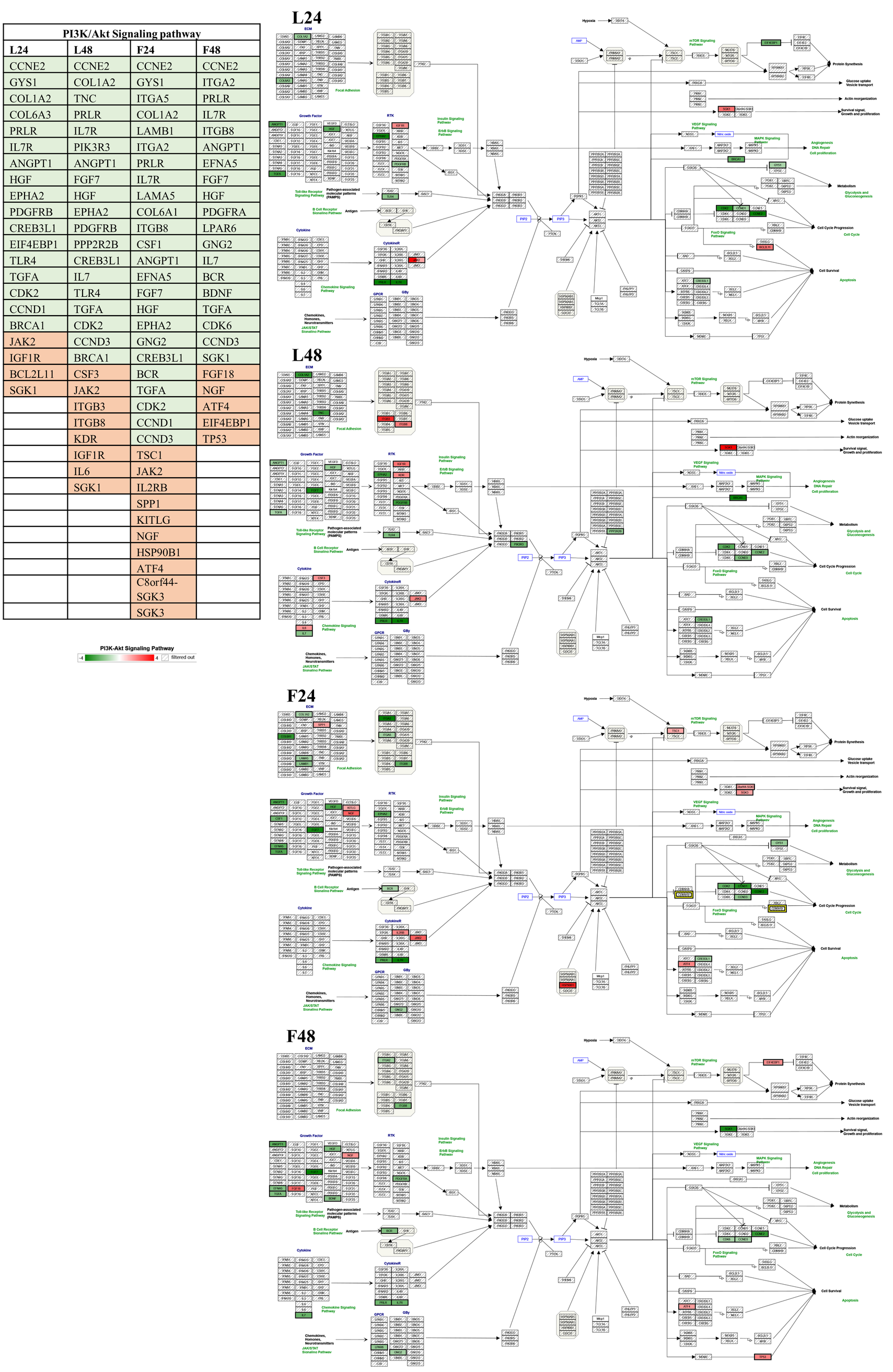

Supplement: S6 Fig — L24, PI3K/Akt signaling pathway affected by LY-294002 at 24h; L48, PI3K/Akt signaling Pathway affected by LY-294002 at 48h. F24, PI3K/Akt signaling Pathway affected by fucoxanthin at 24h. L48, PI3K/Akt signaling Pathway affected by fucoxanthin 48h, showing down-regulated (left tables, in green) and up-regulated genes (left tables in red) in response to individual treatments. (TIF) [file pone.0239551.s010.tif]

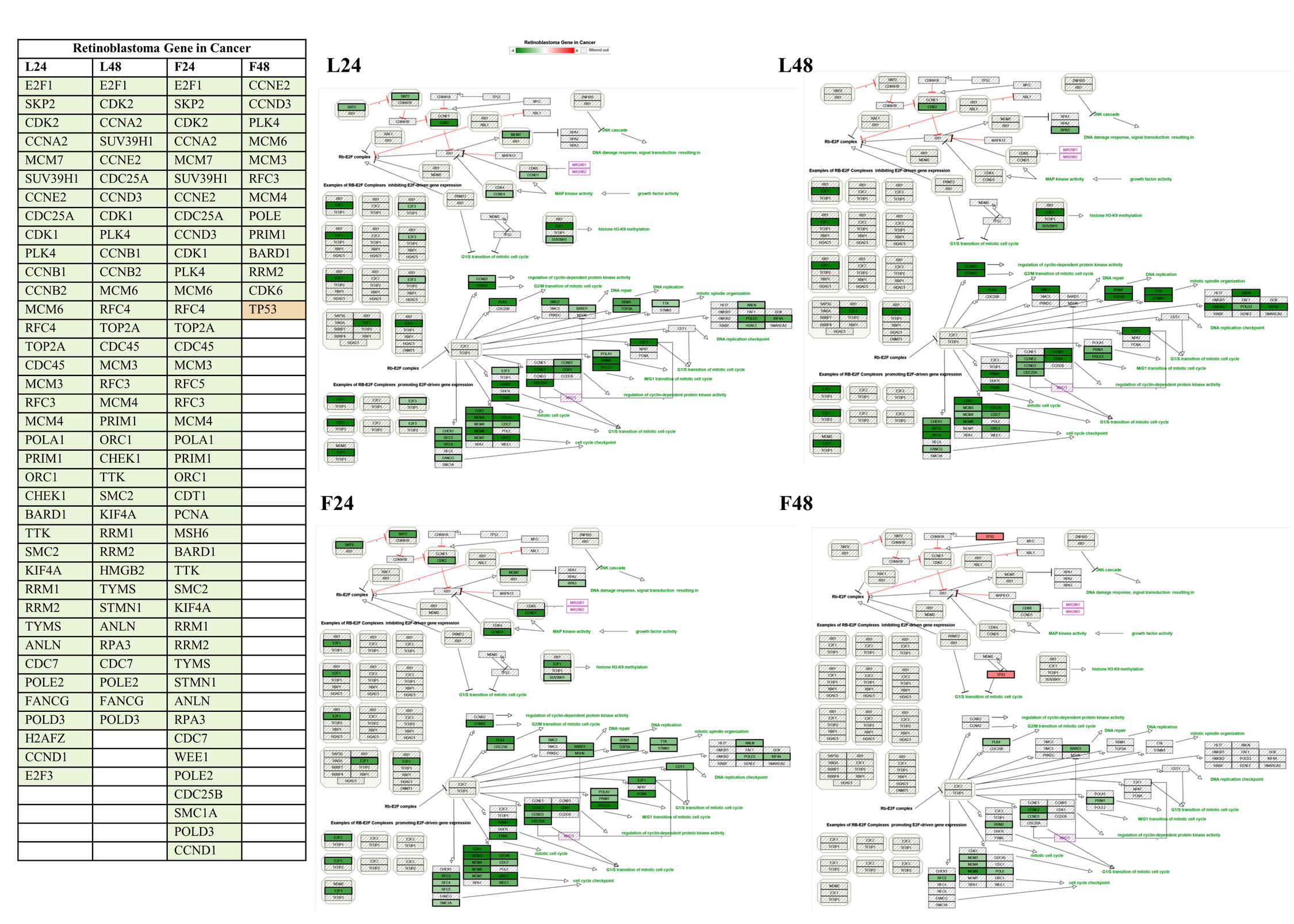

Supplement: S7 Fig — L24, Retinoblastoma gene in cancer pathway affected by LY-294002 at 24h; L48, Retinoblastoma gene in cancer pathway affected by LY-294002 at 48h; F24, Retinoblastoma gene in cancer pathway affected by fucoxanthin at 24h; L48, Retinoblastoma gene in cancer pathway affected by fucoxanthin at 48h; together with the down-regulated (left table in green) and up-regulated genes (left table in red) in response to each individual treatment. It can be seen that the effector end of the pathway is down-regulated the most. (TIF) [file pone.0239551.s011.tif]
